# Modern Perspectives on the Mechanisms and Non-Genetic Factors in Ménière’s Disease—A Narrative Review

**DOI:** 10.3390/biomedicines14051132

**Published:** 2026-05-16

**Authors:** Iustin Mihai Iațentiuc, Otilia Elena Frăsinariu, Andreea Iațentiuc, Lucia Corina Dima-Cozma, Raluca Olariu, Elena Tătăranu, Alexandru Florescu, Andreea Moaleș, Andrei Osman, Mihaela Durnea, Ionuț Daniel Iancu, Sebastian Romică Cozma, Oana Roxana Bitere-Popa

**Affiliations:** 1 Grigore T. Popa University of Medicine and Pharmacy, 700115 Iași, Romania; iatentiuc_iustin@yahoo.com (I.M.I.); andreea.iatentiuc@umfiasi.ro (A.I.); cozma.dima@umfiasi.ro (L.C.D.-C.); raluca.olariu@umfiasi.ro (R.O.); alexandru-florin.florescu@umfiasi.ro (A.F.); andreea.moales@umfiasi.ro (A.M.); mihaela.durnea@umfiasi.ro (M.D.); iancu.ionut-daniel@email.umfiasi.ro (I.D.I.); sebastian.cozma@umfiasi.ro (S.R.C.); oana-roxana.bitere@umfiasi.ro (O.R.B.-P.); 2 Faculty of Medicine and Biological Sciences, “Stefan cel Mare” University of Suceava, 720229 Suceava, Romania; etataranu@gmail.com; 3 Department of Anatomy and Embriology, Faculty of Dentistry, University of Medicine and Pharmacy of Craiova, 200349 Craiova, Romania; andrei.osman@umfcv.ro

**Keywords:** non-genetic factors, Ménière’s disease, pathophysiology, endophenotypes

## Abstract

Ménière’s disease is a complex disorder of the inner ear, characterized by recurrent episodes of vertigo, progressive hearing loss, tinnitus, and aural fullness. The pathophysiology of this condition is dominated by endolymphatic hydrops, reflecting imbalances in fluid regulation and pressure within the membranous labyrinth, which impair cellular function and the transmission of vestibular and auditory signals. Although genetic predisposition provides a susceptible background, recent studies emphasize that non-genetic factors act as critical triggers of clinical events, determining both the onset of symptoms and the modulation of their severity. These factors directly influence pathophysiology by disrupting endolymphatic homeostasis and altering the intracellular and tissue dynamics of the membranous labyrinth, thereby contributing to the phenotypic variability observed among patients. The key to this process lies in the synergistic interaction between genetic predisposition and external or contextual influences, which determines the threshold at which the compensatory mechanisms of the inner ear fail, triggering the characteristic episodes. Understanding this interdependence, as well as the underlying disease mechanisms, provides essential insights for the identification of preventive and therapeutic strategies aimed not only at symptom control but also at modulating the factors that influence susceptibility to endolymphatic imbalance.

## 1. Introduction

At present, the pathophysiology of Ménière’s disease (MD) remains incompletely understood, with multiple theories having been proposed (genetic, anatomical, metabolic, endocrine, autoimmune, and vascular), in association with various extrinsic factors such as allergies, viral infections, psychological stress, or trauma [1,2]. This suggests that endolymphatic hydrops (EH) may represent rather an epiphenomenon, resulting from disruption of the homeostatic balance of the inner ear, which can be triggered by multiple mechanisms. A multifactorial perspective is essential to explain the clinical heterogeneity and variability in disease progression [3,4]. The literature highlights the role of regulatory systems such as innate immunity, the endocrine system, and the autonomic nervous system, which may modulate phenotypic expression in familial forms of Ménière’s disease (FMD) [5]. The involvement of these mechanisms suggests that the final disease phenotype is shaped by the interaction between one or more triggering factors and the individual patient’s response.

Each of these lines of inquiry is supported by varying levels of scientific evidence; however, only a few hypotheses have been consistently substantiated across studies. For example, although some early findings suggested a potential autoimmune involvement, these observations were not subsequently confirmed in a systematic manner, and the immunological role remains unclear, albeit highly plausible. Similarly, metabolic or endocrine theories do not satisfactorily explain why the disease typically presents unilaterally, while allergic and vascular mechanisms suffer from the same conceptual limitation [6].

Although none of the existing theories can be completely ruled out, two have gained particular interest in attempts to explain the predominantly unilateral nature of the disease: migraine and reactivation of latent viral infections. Migraine provides a pathophysiological model in which vascular dysfunction, neuroinflammation, and increased sensitivity of neurosensory structures may contribute to the development of aural symptoms. Conversely, the hypothesis of latent viral reactivation (most discussed in relation to herpes simplex virus) offers a mechanism capable of producing localized inner ear damage, with possible periods of exacerbation and remission [6,7].

Non-genetic factors constitute essential elements in the pathogenesis of MD, influencing susceptibility, disease onset, and clinical progression. These factors act in close interdependence with genetic predisposition, modulating the phenotype through their effects on endolymphatic homeostasis, immunological responses, and vulnerability to inflammation or oxidative stress. Studies suggest that exposure to environmental factors, lifestyle influences, and individual biological conditions may exacerbate symptomatology, helping to explain the episodic recurrence of attacks and the differences observed among individuals who appear genetically similar [2,8].

Understanding of the underlying pathophysiological mechanisms remains limited, as most existing studies have been conducted on small cohorts and heterogeneous populations, which reduces statistical power and the reproducibility of findings. Consequently, although there is consensus regarding the multifactorial nature of the disease, the precise mechanisms leading to its onset and progression remain insufficiently elucidated, highlighting the need for further studies to clarify these processes. This review article aims to highlight the principal mechanisms underlying Ménière’s disease and their relationship with non-genetic factors that contribute to the emergence of distinct endophenotypes. Using PubMed and Scopus, we selected the most relevant studies based on statistical robustness and chronological relevance. Based on the data analyzed, we outline conclusions of value for future research and for the individualized study of the disease. Emphasis should be placed on the prevention of this highly disabling condition, as well as on improving the quality of life of diagnosed patients.

## 2. Clinical Aspects of Ménière’s Disease

MD is one of the most frequently encountered disorders in otorhinolaryngology and audiology, representing a chronic pathology of the inner ear defined by cochlear and vestibular dysfunction. Its clinical manifestations include sensorineural hearing loss, episodic vertigo attacks, tinnitus, and a sensation of aural fullness. Symptoms may fluctuate during active periods, and remission can persist for extended intervals [1,2,9].

The dominant symptom of MD, recognized in nearly all cases, remains recurrent spontaneous vertigo, present in more than 96% of patients [9]. This represents the manifestation with the greatest functional impact, perceived as an intense sensation of environmental rotation, exacerbated by head movements and frequently associated with nausea, vomiting, and diaphoresis. Vertigo attacks have an episodic onset and typically last several hours, with recovery often followed by a period of postural instability that may persist for several consecutive days. In many cases, these episodes are preceded by an exacerbation of auditory symptoms, including tinnitus, aural fullness, and temporary hearing deterioration on the affected side [9,10].

In clinical practice, the most common etiologies of episodic vertigo are benign paroxysmal positional vertigo and vestibular migraine [11,12]. However, vertiginous episodes in MD are typically of longer duration and are not reproducible by specific head movements, a characteristic that facilitates their differentiation [13]. Additionally, sensations of imbalance or dizziness without a perception of environmental rotation are frequently encountered in orthostatic hypotension, most often because of dehydration or cardiogenic causes [8,14]. Cochlear otosclerosis should also be considered in the differential diagnosis, as it may generate vestibular symptoms in approximately 30–35% of patients [15,16]. Although less frequently confused with MD, acute vestibular neuritis may reproduce some of its manifestations; however, episodes are generally of longer duration and are not accompanied by auditory symptoms, which clearly distinguish between the two conditions [17,18].

Hearing loss is associated with vertigo episodes in 77% of cases [19]. Initially, it fluctuates (i.e., reversible after an attack) and most frequently affects low frequencies. However, as the disease progresses, hearing progressively deteriorates and no longer fully recovers following attacks. Eventually, permanent deafness may occur. Some patients report a prior history of hearing loss (since childhood) that precedes the onset of vertigo. This variant is referred to as delayed Ménière’s disease [9,20].

Tinnitus may be the initial symptom, preceding the full clinical picture by several months. It is often described as a low-pitched, roaring sound, like the noise of machinery or a seashell. At onset, it is intermittent and occurs during attacks in 83% of patients, subsequently resolving [19,20]. As attacks recur, tinnitus becomes persistent between episodes, although its intensity or pitch may fluctuate before or during an attack, serving as a warning signal for patients. In advanced stages, when vertigo subsides, tinnitus becomes the dominant symptom and significantly affects quality of life [9].

Differentiation of MD from other conditions that may cause tinnitus is essential, including metabolic disorders, sleep disturbances, adverse reactions to certain medications, or psychological factors, all of which may contribute to a similar clinical presentation but with a different underlying etiology [8]. The study conducted by Tyrrell et al. highlights two key clinical features of MD: increased tinnitus severity and a significantly higher risk of falls [3]. Both have important implications for patient management. Comparative analysis shows that individuals diagnosed with MD experience more intense and distressing tinnitus than those in the control group, suggesting a substantial impact on psychological well-being and quality of life. Given the intrusive nature of tinnitus and its association with sleep disturbances, anxiety, and depression, these findings underscore the need for targeted therapeutic interventions aimed at reducing auditory symptom severity and mitigating its emotional consequences.

Aural fullness is variable, with more than 20% of patients not experiencing this symptom at all [20]. It is described as a sensation of pressure in the ear, like that experienced during airplane landing. It usually diminishes as the disease progresses. Other reported symptoms include hyperacusis (increased sensitivity to sound) and diplacusis (perception of the same sound at different pitches in the two ears). Approximately 68% of patients experience at least two of the three auditory symptoms (unilateral hearing loss, unilateral tinnitus, unilateral aural fullness) during at least half of vertigo attacks [9].

Some patients also experience abrupt falls occurring without prior vertigo, loss of consciousness, or other neurological manifestations. These events, referred to as Tumarkin otolithic crises, reflect an abrupt dysfunction of the otolithic mechanisms responsible for balance control. Although their prevalence is variable, some studies report high rates, affecting up to 72% of patients. The presence of these episodes is clinically significant, as it increases the risk of trauma and adds to the functional burden of the disease [10,21,22].

Tyrrell et al. further report a twofold increase in fall risk among patients with MD compared with controls, marking the first investigation to quantify this difference within a well-defined control group. This finding confirms the significant impairment of vestibular function and postural balance characteristic of the disease, while also drawing attention to its physical and socioeconomic consequences. The increased risk of falls not only heightens patients’ physical vulnerability but may also contribute to deteriorating mental health and rising healthcare costs, representing a major public health concern [3].

In most cases, MD symptomatology initially presents unilaterally, and after several years, tinnitus and hearing loss also develop in the contralateral ear. However, rare cases exist in which patients present with bilateral sensorineural hearing loss from disease onset. In unilateral forms, the presence of tinnitus and aural fullness in the affected ear is required to confirm the diagnosis [8].

MD is characterized by marked clinical heterogeneity, with the course of vertigo episodes and hearing loss varying considerably from one patient to another. In addition, the accurate identification of familial cases is complicated by the fact that disease onset almost always occurs in adulthood [23]. FMD represents a clinically variable condition, and some individuals who exhibit only partial clinical features (such as vertigo episodes without low-frequency sensorineural hearing loss) may not meet the criteria required for a diagnosis of MD [5,8].

The auditory symptoms of MD are closely linked to the pathophysiological changes induced by EH. An increase in endolymphatic volume leads to distension of the membranous labyrinth, with a direct impact on cochlear mechanics and the function of the organ of Corti. In particular, the apical region of the cochlea, which is responsible for the perception of low frequencies, is most susceptible to variations in endolymphatic pressure, explaining the early involvement of hearing in this frequency range. Fluctuations in endolymphatic volume result in transient changes in the endocochlear potential and mechanoelectrical transduction, which clinically translate into the variable and reversible nature of hearing loss in the early stages of the disease. Tinnitus and the sensation of aural pressure can be explained by abnormal activation of sensory cells and afferent nerve fibers, secondary to pressure-related and ionic changes within the endolymph. However, the discrepancy between the severity of hydrops and the intensity of symptoms observed in some post-mortem studies suggests that hydrops is necessary but not sufficient for the development of clinical manifestations, acting rather as a pathophysiological mediator than as the sole cause of the disease [24,25].

Episodic vertigo results from acute disruption of vestibular function generated by dynamic changes in the endolymphatic environment. Sudden distension of vestibular structures may alter the excitability of sensory cells within the maculae and ampullary cristae, leading to asymmetric discharge of vestibular afferents. In some pathophysiological models, transient rupture of Reissner’s membrane, with mixing of potassium-rich endolymph and perilymph, causes abrupt and dysfunctional depolarization of vestibular receptors, explaining the violent and self-limiting nature of vertigo attacks. Repetition of these episodes over time leads to cumulative damage to sensory epithelia and progressive vestibular hypofunction, accounting for the clinical transition from paroxysmal vertigo to chronic instability. Thus, pathophysiological mechanisms not only trigger vestibular symptoms but also shape their evolution over time, conferring MD its characteristic fluctuating and progressive course [13,24].

The symptoms of MD directly reflect the activity of pathophysiological mechanisms that disrupt inner ear homeostasis. Understanding this relationship is essential, as symptoms represent indicators of ongoing pathological processes, and their correct interpretation provides the basis for diagnostic and therapeutic approaches directed toward the underlying mechanisms, rather than solely toward symptomatic disease control.

## 3. Diagnosis and Clinical Course

**Figure 1:**
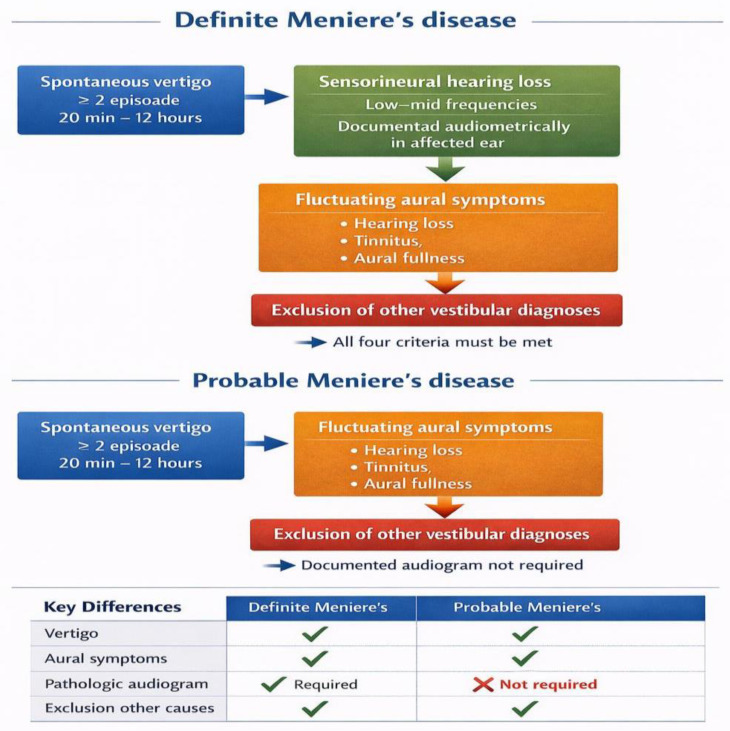
The classification of Ménière’s disease.

The first attempts to standardize the diagnosis of MD were undertaken by the American Academy of Otolaryngology–Head and Neck Surgery (AAO-HNS), which published diagnostic guidelines in 1972 and 1985, and subsequently, in 1995, established the first internationally accepted diagnostic criteria [26]. The diagnostic criteria for MD were later defined through an international consensus involving major professional organizations, including the Classification Committee of the Bárány Society, the Japan Society for Equilibrium Research, the European Academy of Otology and Neurotology (EAONO), the Balance Committee of the American Academy of Otolaryngology–Head and Neck Surgery (AAO-HNS), and the Korean Balance Society. As a result of this consensus, MD is classified into two main clinical forms: definite Ménière’s disease and probable Ménière’s disease [9]. This classification allows improved diagnostic standardization and facilitates the early identification of patients at risk of developing the definite form of the disease (Figure 1).

Because in most patients the clinical picture is not fully established during the early stages of the disorder, the diagnosis is generally made only several months or even years after the onset of initial manifestations [27,28]. A definite diagnosis of MD is established when the patient presents at least two typical episodes of vertigo, each lasting more than 20 min, associated with fluctuating sensorineural hearing loss documented by serial audiograms, along with the presence of tinnitus and/or aural fullness in the affected ear. Exclusion of alternative causes of vertigo is essential prior to diagnostic confirmation [1]. Thereafter, diagnosis relies primarily on clinical presentation and audiometric testing, as no specific biomarkers are currently available to confirm the disease. Clinical manifestations evolve over time: vertigo attacks are intermittent and vary in frequency and intensity, with durations ranging from 20 min to 12 h, while hearing loss, initially fluctuating, becomes progressively irreversible as the disease advances. Sensorineural hearing loss predominantly affects low or mid frequencies [8,9].

The onset of MD is often insidious and variable, making early recognition of the condition challenging in clinical practice. Only approximately 40% of patients present with the classic triad at disease onset, comprising recurrent episodes of vertigo, fluctuating sensorineural hearing loss, and ipsilateral tinnitus [23]. In most cases, the disease begins with incomplete symptoms, which may be nonspecific and mimic other vestibular or auditory disorders [9].

It is possible for recurrent episodes of isolated vertigo, without associated auditory symptoms, or fluctuating hearing loss, with or without tinnitus, to precede the development of the full clinical picture by months or even years. This fragmented nature of onset contributes to diagnostic delays and confusion with other clinical entities. Longitudinal studies have shown that in approximately 20% of patients, the interval between the onset of hearing loss and the occurrence of the first vertigo attacks may exceed five years. Nevertheless, in most patients, the complete triad becomes apparent within the first year of disease progression [9,27].

The clinical course of MD is characterized by fluctuations in symptom intensity and frequency. Vertigo episodes are generally the most disabling manifestations during the early stages of the disease, significantly impacting quality of life. Over time, the frequency of these episodes tends to decrease spontaneously. Observational studies have demonstrated that approximately 70% of patients who remain free of vertigo for one year continue to be asymptomatic in the subsequent year [9,29].

Long-term analyses indicate that the frequency of vertigo attacks declines rapidly during the first 8–9 years of disease progression, followed by a period of stabilization and, subsequently, a gradual reduction in vestibular symptoms. This trajectory suggests a possible progressive “exhaustion” of vestibular function, which may lead to amelioration of vertigo but is often accompanied by worsening hearing loss and the development of chronic imbalance [9].

## 4. Classification and Endophenotypes

The heterogeneity of clinical manifestations has represented a major challenge for genetic studies of MD. However, this symptomatic variability may reflect the presence of genetic differences that influence distinct biological processes, thereby generating diverse endophenotypes within the same disease. Initially, two clinical forms were identified based on auditory involvement: unilateral and bilateral forms [8]. Consequently, numerous studies have focused on establishing clear classifications to improve understanding of the disease and to differentiate patient categories according to their presenting symptoms. For instance, the study conducted by Frejo et al. identified a series of clinical variables capable of dividing patients into five distinct subgroups [30]. A subsequent study, which included 1073 patients with unilateral MD, also conducted by Frejo et al. [28], identified several subgroups, slightly different from the initial findings. Based on the conclusions of these studies, Table 1 was developed to summarize the main endophenotypes of MD.

The table compares two classifications of patients with MD into distinct groups, defined based on disease onset, hearing loss distribution, the presence of migraine, autoimmune comorbidities, and family history. Although the percentages differ slightly between the two columns, the overall structure is similar and indicates the existence of recurring, reproducible clinical subtypes. The table demonstrates that MD is not a single clinical entity, but rather a spectrum of distinct endophenotypes, each likely associated with different pathogenic mechanisms. Classifying patients according to these endophenotypes allows for a reduction in heterogeneity in genetic and epidemiological studies; provides an explanation for contradictory findings across populations and studies; facilitates the identification of biomarkers specific to each subtype; and supports the development of personalized therapeutic strategies. Thus, endophenotypes represent an essential conceptual tool for transitioning from a descriptive view of MD to a mechanistic and predictive perspective, with direct implications for both research and clinical practice.

The identification and categorization of patients into specific clinical subgroups constitute a critical step in the assessment and diagnosis of MD. This approach provides an initial framework for understanding MD not merely as a single disorder but as a core phenotype that may be associated with a variety of clinical manifestations and comorbid conditions. Clinical subgroups, such as unilateral or bilateral MD, patterns of hearing loss, and the presence of episodic or continuous vertigo and tinnitus, offer valuable insights into underlying pathophysiological mechanisms and potential disease trajectories. In clinical evaluation, it is also essential to consider associated comorbidities, which appear to significantly influence both disease onset and progression [8,28].

It is also noteworthy to consider findings from radiological studies suggesting that alterations of the endolymphatic sac may correspond to distinct clinical subtypes of MD. Using gadolinium-enhanced magnetic resonance imaging (MRI), two distinct endotypes of patients with MD have been described, differentiated based on endolymphatic sac morphology. The first endotype is characterized by a predominantly degenerative process of the endolymphatic sac epithelium, suggesting progressive loss of its regulatory function in controlling endolymphatic volume. The second endotype is defined by endolymphatic sac hypoplasia, a developmental anomaly indicative of a structurally reduced capacity to maintain endolymphatic homeostasis [8,31].

The hypoplastic endotype demonstrated a strong correlation between the presence of ipsilateral EH and the clinical manifestations of MD. However, no clinical or radiological changes were observed to suggest involvement of the contralateral ear. Consequently, patients classified within this endotype present a lower risk of developing MD in the opposite ear compared with individuals assigned to the degenerative endotype. The degenerative endotype, which is more commonly observed, was primarily associated with the occurrence of contralateral EH in the absence of clinical symptoms indicative of inner ear dysfunction. These “silent” forms of hydrops were observed in 19.2% of patients with unilateral MD within this endotype. This proportion is consistent with previously reported frequencies in other MRI studies of patients with unilateral MD, suggesting a reproducible phenomenon that may be characteristic of this subpopulation [8,31].

Although it has been hypothesized that individuals with the degenerative endotype may theoretically have a higher risk of progression to a bilateral form, such progression has been rarely documented. Nevertheless, patients in this category exhibited more frequent vertigo episodes and a greater degree of vestibular impairment, which may reflect more pronounced functional instability of the vestibular system in the context of degeneration [8].

Therefore, understanding MD requires an integrative perspective that combines detailed clinical evaluation, analysis of comorbidities, and investigation of the genetic and immunological mechanisms involved. Only through a holistic approach can earlier diagnosis, precise patient stratification, and optimized treatment be achieved, minimizing long-term impacts on patients’ quality of life. This complexity of the MD phenotype underscores the importance of future studies involving large, well-characterized populations to elucidate the relationships between genes, environment, and the pathological mechanisms underlying the disease.

## 5. Non-Genetic Factors and Pathophysiological Mechanisms

**Figure 2:**
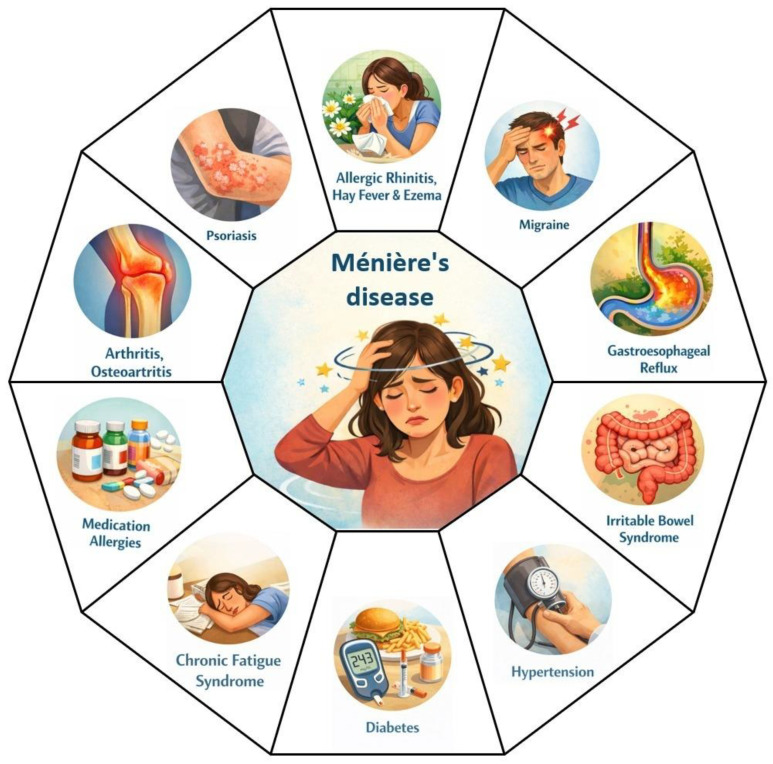
Diseases that may be associated with Ménière’s disease.

Ménière’s disease is a complex disorder of the inner ear whose pathogenesis cannot be explained solely by genetic determinism. In addition to hereditary susceptibility, a variety of non-genetic factors play a critical role in initiating, triggering, and modulating the clinical course of the disease. Through diverse mechanisms, these factors can disrupt inner ear homeostasis, particularly the regulation of endolymphatic volume and composition, contributing to the development of EH and characteristic symptoms. Various categories of factors appear to precipitate symptomatic episodes in MD. These include psychological stress, sleep deprivation, certain types of food, exposure to allergens, individual characteristics (such as age, sex, and race), socio-economic and environmental factors, viral infections, as well as hormonal changes. Although the precise mechanisms by which these factors influence the onset of attacks are not yet fully elucidated, the literature suggests that they may destabilize inner ear homeostasis in predisposed individuals [9]. Moreover, Ménière’s disease can be caused by or associated with a wide range of disorders, which may influence its severity, onset, as well as the pathophysiological mechanisms through which it is triggered [9] (Figure 2).

The importance of non-genetic factors and the mechanisms they trigger is underscored by the fluctuating nature of MD. These factors often act as triggers or amplifiers of the disease on a genetically predisposed background, illustrating the multifactorial nature of the condition. In this context, understanding the role of non-genetic factors is essential not only for clarifying pathogenic mechanisms but also for the development of personalized therapeutic and preventive strategies that target not only the genetic component but also the environmental and systemic influences associated with MD.

A critical aspect that must be emphasized is the significant heterogeneity of studies investigating the role of non-genetic factors in Ménière’s disease. Most of the available data derive from observational, retrospective studies or case series, which limits the ability to establish direct causal relationships between exposures and the described pathophysiological mechanisms. Furthermore, variability in diagnostic criteria used prior to the standardization proposed by the Bárány Society introduces an additional risk of including non-homogeneous populations, particularly in the context of overlap with vestibular migraine or other vestibular disorders. This may lead to overestimation or underestimation of the role of certain factors such as migraine, allergies, or sleep disturbances.

### 5.1. Endolymphatic Hydrop Theory

The primary pathological change observed in MD is EH, which consists of dilation of the endolymphatic compartment due to excessive accumulation of endolymph within the scala media and the endolymphatic sac of the inner ear [4]. This accumulation disrupts the normal balance between endolymph and perilymph and affects the mechanical and ionic mechanisms essential for auditory and vestibular transduction. The development of EH reflects an imbalance between endolymph production, circulation, and reabsorption. The inner ear possesses precise mechanisms for regulating both the volume and chemical composition of endolymph, with the endolymphatic sac and duct serving as key structures in maintaining this equilibrium [24,25].

Dysregulation of osmotic pressure and electrochemical gradients impairs the function of sensory and supporting cells, suggesting that EH is the result of an active, dynamic process rather than a simple passive obstruction. Experimental and clinical studies support the notion that disruption of endolymphatic homeostasis is a multifactorial process influenced by vascular, inflammatory, and metabolic mechanisms [25]. The hypothesis of an imbalance in ionic homeostasis, particularly in the Na^+^/K^+^ ratio, remains plausible given the fragility of electrolyte circuits within the cochlea and their sensitivity to minor alterations in the endolymphatic microenvironment. However, current evidence does not allow for the identification of a single mechanism or a definitive triggering factor [6,32].

Initially, EH was considered the defining feature of MD [33]; however, subsequent temporal bone studies have demonstrated the presence of EH not only in patients with MD but also in asymptomatic individuals, as well as in the apparently normal contralateral ear of affected patients, suggesting that EH alone cannot account for symptom onset [1,34,35,36]. Furthermore, cases of MD without EH have been described, indicating that EH is neither essential nor specific to the disease [1]. A recent analysis confirms that the relationship between MD and EH remains unclear: hydrops may contribute to symptom development, but in the absence of additional factors, it is insufficient to trigger the disease [2,25]. Limitations in the in vivo exploration of the sensitive structures of the inner ear, along with only recent advances in imaging techniques, have rendered MD a difficult condition to study [35].

The causes leading to the development of EH are heterogeneous and reflect the interaction between individual susceptibility and triggering factors [37]. Altered endolymphatic sac function, changes in vascular permeability, and local immunological imbalances may contribute to the loss of control over endolymph volume. In this context, EH is considered the final common expression of diverse etiopathogenic mechanisms, which explains the clinical and evolutionary variability of MD [38]. Among the main causes of EH are viral infections, autoimmune or autoinflammatory processes, as well as genetic factors associated with developmental anomalies, such as temporal bone hypoplasia [8,39].

Taken together, the available evidence supports an integrative view of MD pathogenesis, in which EH represents only one of multiple consequences of broader homeostatic dysfunction, and individual susceptibility plays a central role in determining how the disease manifests and progresses.

### 5.2. Ménière’s Disease and Migraine

Up to 56% of patients diagnosed with MD also experience migraine, suggesting a possible shared pathogenic pathway between the two conditions [3,40,41]. This clinical association has led to the hypothesis that episodic vertigo, migraine, and the characteristic symptoms of MD (fluctuating sensorineural hearing loss, tinnitus, and aural fullness) may represent different manifestations of a single clinical spectrum [1]. The findings of Radtke et al., showing that approximately half of patients with MD meet clinical criteria suggestive of migraine, support the concept of a shared vulnerability, potentially related to vascular regulatory mechanisms, neuroinflammation, or genetic susceptibility [40,42]. Cha et al. further emphasized that the concurrent occurrence of these symptoms among close relatives and even monozygotic twins supports the existence of a “migraine–Ménière syndrome” with variable phenotypic expression [43].

One of the most important mechanisms linking migraine and MD is neurovascular dysfunction. In migraines, activation of the trigeminovascular system leads to the release of vasoactive neuropeptides, resulting in alterations of vascular tone. The inner ear, supplied by a terminal circulation lacking effective collateral pathways, is particularly vulnerable to vasospasm and fluctuations in perfusion [44]. Recurrent episodes of transient ischemia may impair the function of secretory epithelia and of structures involved in endolymph regulation, thereby promoting the development or exacerbation of EH. This vascular vulnerability provides a plausible explanation for both the episodic nature of symptoms and their association with migrainous phenomena [45]. Within the inner ear, this increased permeability may disrupt the blood–labyrinth barrier, altering the ionic and osmotic balance of the endolymph. This mechanism supports the concept that migraine may act as a functional trigger of Ménière-like symptoms, without the necessity of a permanent structural lesion [40,46].

Migraine is characterized by central neuronal hyperexcitability and abnormal processing of sensory stimuli. This may affect the way vestibular and auditory information is centrally integrated. In the context of MD, peripheral dysfunction induced by EH may be amplified by migrainous central mechanisms, leading to an enhanced perception of vertigo, tinnitus, and auditory discomfort. Thus, migraine not only coexists with MD but may also modulate its clinical expression, contributing to increased symptom severity and frequency [47].

Baloh and Andrews described among the earliest the role of cortical spreading depression, a central mechanism implicated in migraine. This phenomenon is characterized by rapid changes in ionic concentrations, cellular edema, and a transient inhibition of cerebral electrical activity. Although these processes do not faithfully reproduce the mechanisms of EH, they exhibit relevant similarities that may contribute to the understanding of shared mechanisms between migraine and MD and represented some of the earliest evidence supporting this association [48]. Morrison et al. observed, in a cohort of 190 patients with MD, a prevalence of 33% for classic migraine and 30% for a family history of migraine [49]. In a subsequent extended study, 47 of 118 families with MD (40%) exhibited migraine, and among these 47 clearly affected families, more than half had a positive family history [48]. This degree of familial aggregation suggests a potential genetic relationship between the two conditions. Although the cited studies are relatively old, they remain highly relevant and provide an important foundation upon which more complex and comprehensive investigations can be developed.

Migraine has a well-established genetic component, and advances in molecular genetics have identified multiple chromosomal regions involved in susceptibility to the disorder. To date, at least nine genetic loci have been associated with common migraine (with or without aura) and three with familial hemiplegic migraine (FHM). In FHM, mutations have been identified in three key genes (CACNA1A, ATP1A2, and SCN1A), all of which encode ion channels. The molecular defects described affect intracellular ionic homeostasis, leading to rapid alterations in electrolyte concentrations. This mechanism is particularly compelling when attempting to explain episodic vertigo and fluctuating hearing loss in MD, as the inner ear is extremely sensitive to ionic variations, and calcium, sodium, and potassium channels play a crucial role in the function of both the cochlea and the vestibular system [6,50].

Differentiating MD from migraine based solely on current clinical criteria remains challenging. In some cases, particularly in women approaching menopause, migraine may be accompanied by episodes of vertigo that closely mimic the symptoms of MD. Vestibular migraine occurs significantly more frequently in women [20]. A genomic study conducted in a large multigenerational family spanning four affected generations identified a potential susceptibility locus on chromosome 5q35, located between the markers rs244895 and D5S2073. This genomic region contains genes involved in the regulation of ionic transport (KCNMB1, KCNIP1, ATP6V0E, SLC34A1), as well as genes related to neuronal signal transmission (GABRP, DRD1, HRH2). Although these candidates genes differ from those previously associated with FMD, they may also play a role in its pathogenesis, given that the inner ear is rich in ion channels, transporters, and neurotransmitter receptors [4,41,51]. The higher prevalence of migraine among patients with FMD suggests that certain genetic mutations may contribute simultaneously to both conditions. Consequently, migraine may represent a partial phenotype of FMD or, conversely, MD may constitute a specific manifestation of a genetic predisposition to migraine [4].

Aquaporins, particularly AQP4, may represent a common mechanism linking migraine and Ménière’s disease through their role in regulating fluid homeostasis. In the inner ear, AQP1, AQP4, and AQP5 are involved in the control of endolymphatic volume, and their dysregulation has been associated with the development of EH. In parallel, in migraine, AQP4 contributes to the regulation of cerebral water balance and to the propagation of cortical spreading depression, a central mechanism of the disease. Thus, aquaporin dysfunction may promote both peripheral fluid imbalance in MD and central neuronal hyperexcitability in migraine, suggesting a shared pathophysiological substrate. However, direct evidence remains limited, and most of the available data derive from experimental studies [41,42,45].

Migraine is more prevalent among Caucasians, exhibits familial aggregation, and familial hemiplegic forms follow an autosomal dominant (AD) inheritance pattern with incomplete penetrance [4]. Similar characteristics are observed in FMD, including predominantly maternal transmission, although notable sex-related differences in prevalence and incidence persist. In addition, migraine and MD share common triggering factors, such as psychological stress and hormonal fluctuations. Advances in migraine genetics are therefore highly relevant to the understanding of FMD. Genetic heterogeneity is evident even in familial hemiplegic migraine, which has been mapped to chromosomes 19p13 (between D19S216 and D19S215) and 1q31 (between D1S158 and D1S2781), while linkage studies for common migraine, with or without aura, have yielded inconclusive results [43,48,50].

Data from the UK Biobank confirm a significant association between MD, female sex, and the presence of migraine, raising the hypothesis that a subset of cases diagnosed as Ménière disease may in fact correspond to a vestibular migraine phenotype. This observation underscores the need to refine diagnostic criteria and to develop biomarkers capable of reliably differentiating between these two clinical entities [3].

From a clinical perspective, MD and vestibular migraine may exhibit substantial overlap, particularly regarding recurrent episodes of vertigo; however, their overall symptom profiles differ in essential ways. MD is characterized by progressive and fluctuating auditory impairment, predominantly affecting low frequencies in the early stages, and is associated with persistent tinnitus and aural fullness. These auditory manifestations are defining features of the disease and tend to worsen over time, reflecting progressive deterioration of cochlear function. In contrast, vestibular migraine is rarely associated with objective, progressive hearing loss, and when auditory symptoms are present, they are typically transient and nonspecific. Vertigo in vestibular migraine is often variable in duration and intensity and may occur independently of headache, but it is frequently accompanied by photophobia, phonophobia, or other central migrainous features. This distinction in the nature and temporal evolution of auditory symptoms represents one of the most important clinical elements for differentiating between the two entities. This parallel is particularly noteworthy given that both conditions involve paroxysmal episodes, share similar triggers, and display a fluctuating course of symptoms [6,7,12,19,20,52]. Accurate differentiation between these two disorders is essential, as their therapeutic and prognostic implications differ substantially, and overdiagnosis of MD may lead to unnecessary or inappropriate interventions.

Therefore, the association between MD and migraine should not be regarded merely as a clinical coincidence but rather as indicative of shared pathophysiological mechanisms, with important implications for both diseases understanding and the development of personalized therapeutic strategies. From a therapeutic standpoint, agents commonly used in migraine prophylaxis, such as beta-blockers, calcium channel blockers, or antiepileptic drugs, have been shown to alleviate symptoms in some patients with MD, suggesting an overlap in the underlying pathophysiological mechanisms of the two conditions [53,54,55]. This observation opens the perspective for the use of shared therapeutic strategies, particularly in patients with refractory forms of MD.

### 5.3. Ménière’s Disease and the Role of Autoimmunity

From an epidemiological perspective, MD is frequently associated with a range of autoimmune and allergic conditions, including rheumatoid arthritis, allergic rhinitis, systemic lupus erythematosus, allergic dermatitis, ankylosing spondylitis, asthma, as well as autoimmune thyroid disorders. These observations suggest that a subset of patients with MD exhibits an underlying dysregulation of immune function. However, the influence of population-specific factors, whether genetic or environmental, remains insufficiently explored and may account for the geographic variability observed in the clinical manifestations and pathophysiology of the disease [3,56,57,58].

Support for the involvement of autoimmunity in MD derives primarily from the favorable therapeutic response observed in some patients treated with systemic or intratympanic corticosteroids. The efficacy of corticosteroid therapy suggests the presence of an active inflammatory process that can be modulated through the inhibition of proinflammatory pathways [59]. Secondly, serological studies have demonstrated the presence of autoantibodies or circulating immune complexes directed against inner ear antigens, indicating aberrant activation of adaptive immunity in a subset of patients with MD [60]. These immune complexes may contribute to tissue damage through complement activation and the recruitment of inflammatory cells.

The autoimmune hypothesis in MD is further supported by the identification of elevated levels of autoantibodies and circulating immune complexes in patient sera, suggesting abnormal immune system activation [1]. Various autoantibodies have been described, including antinuclear antibodies and antiphospholipid antibodies; however, these findings remain inconsistent due to small sample sizes and the lack of replication in larger studies [61,62,63]. Several shared immunological mechanisms have been proposed, including the activation of autoreactive T lymphocytes, excessive production of proinflammatory cytokines, and impairment of immune tolerance mechanisms. Consequently, MD may be viewed not only as an isolated disorder of the inner ear but also as part of a broader spectrum of autoimmune diseases [1].

A central mechanism proposed in autoimmune-mediated MD is the loss of immunological tolerance to self-antigens of the inner ear or to normally harmless stimuli, such as dietary antigens. Under physiological conditions, labyrinthine structures are partially immunoprotected by the blood–labyrinth barrier and by mechanisms of peripheral immune tolerance. When these protective mechanisms are compromised, cochleovestibular antigens may be recognized as non-self, leading to the activation of autoreactive T lymphocytes. These cells can infiltrate inner ear structures and initiate a local inflammatory response, resulting in damage to sensory epithelia and to the structures involved in endolymph regulation. Histopathological and experimental studies have demonstrated the presence of lymphocytic infiltrates and markers of immune activation within the endolymphatic sac, supporting its role as an immunologically active organ of the inner ear. This type of response, exacerbated by genetic predisposition, may lead to a state of chronic low-grade inflammation characterized by persistent immune cell activation and sustained release of inflammatory mediators. Over time, such chronic inflammation may promote progressive deterioration of auditory and vestibular function without producing overt systemic signs of autoimmune disease [56,63,64].

A second major mechanism involves the role of proinflammatory cytokines. Immune activation is accompanied by increased production of cytokines such as TNF-α, IL-1β, and IL-6, which may exert direct effects on inner ear physiology. These molecules influence vascular permeability, the integrity of the blood–labyrinth barrier, and the function of epithelial cells involved in ionic transport. Increased vascular permeability promotes the extravasation of fluids and plasma proteins, disrupting osmotic balance and contributing to endolymph accumulation. Concurrently, cytokines may impair mitochondrial function and sensory cell survival, thereby amplifying auditory and vestibular dysfunction [9,64,65,66,67,68].

A critical step in the initiation of autoimmune processes within the inner ear is the disruption of the blood–labyrinth barrier. Under normal conditions, this barrier restricts the access of immune cells and inflammatory mediators to the sensitive labyrinthine structures. Systemic or local inflammation can compromise this barrier, allowing activated lymphocytes and cytokines to enter the endolymphatic space. Once such access is facilitated, the immune response may become self-sustaining, leading to chronic inflammation and structural dysfunction. This framework helps explain why MD frequently occurs in the context of systemic autoimmune diseases and why disease activity may fluctuate in parallel with overall immunological activity [56,64].

The endolymphatic sac is attributed to a central role in the relationship between MD and autoimmunity. It contains antigen-presenting cells and participates in the immune clearance of endolymph. Dysfunction of the endolymphatic sac, induced by inflammation or direct immune-mediated injury, may impair its capacity to regulate endolymphatic volume and promote the development of EH. Thus, immune mechanisms not only induce inflammation but also directly interfere with the physiological processes responsible for maintaining fluid homeostasis within the inner ear [3].

Another proposed mechanism involves cross-reactive immune responses. According to this hypothesis, antibodies generated in response to viral or bacterial infections recognize similar epitopes present on inner ear structures. This molecular mimicry may result in erroneous immune attacks against the sensory epithelium, the stria vascularis, or the endolymphatic sac, thereby triggering local inflammation and tissue dysfunction. Consequently, infections may act as initiating factors for a persistent autoimmune response [9,69].

Although the autoimmune hypothesis has not yet been conclusively demonstrated experimentally, indirect evidence supports the existence of a subgroup of patients with MD associated with autoimmune mechanisms. In experimental models, autoimmune EH has been induced in laboratory animals through the injection of antigens or monoclonal antibodies, leading to inflammation of inner ear structures. Furthermore, antibodies against type II collagen (a structural component of the inner ear extracellular matrix) have been detected in the serum of patients with MD, suggesting a potential autoimmune process directed against self-tissue components [8,70]. Other studies have reported the presence of circulating autoantibodies in patients with MD; however, it remains unclear whether these antibodies represent a consequence of chronic inner ear inflammation or whether they were present prior to disease onset and thus acted as causal factors [8,61,62,71].

The concept that the immune system may play a role in the etiology of MD was initially highlighted by studies reporting associations between the disease and class II human leukocyte antigen (HLA) alleles, which are involved in antigen presentation and autoimmunity [72,73]. Some investigations have described associations between specific HLA alleles and bilateral MD, as well as between variants of the PTPN22 gene (known to confer susceptibility to systemic autoimmune diseases) and MD [74]. However, these findings have not been consistently replicated in independent cohorts, possibly due to selection bias and population stratification, which may lead to false-positive results in case–control genetic studies [8].

Allelic variants in genes such as MICA (major histocompatibility complex class I-related genes involved in natural killer cell and T lymphocyte activation) [63], TLR10 (encoding a Toll-like receptor involved in pathogen recognition) [75] and NFKB1 (a regulator of proinflammatory gene expression) [76] have been associated with differences in the progression of hearing loss in patients with sporadic MD (SMD). In addition, an association has been reported with a functional variant of lymphoid tyrosine phosphatase (LYP), which inhibits T-cell receptor activation and may contribute to immune dysregulation in patients with bilateral inner ear involvement [1,77].

A subset of patients exhibits elevated levels of proinflammatory cytokines, and exposure to allergens such as Aspergillus and Penicillium elicits an exaggerated inflammatory response, suggesting the involvement of TNF-α- and IL-1β-mediated pathways [78]. Moreover, bilateral MD has been associated with a common regulatory variant (rs4947296) in a large case–control study, highlighting a potential role for NF-κB–dependent inflammation [58,79]. The multifunctional cytokine TWEAK and its receptor Fn14, which modulate activation of the NF-κB signaling pathway (a key transcription factor in inflammatory and autoimmune processes) have been implicated in the regulation of autoinflammation in various systemic diseases [8].

Although autoimmune features have been identified in both unilateral and bilateral forms of MD, the precise nature of the inflammatory mechanism and its distribution across different clinical phenotypes remain poorly defined [28,30]. Some investigations have identified a distinct subgroup of patients characterized by markedly elevated baseline levels of proinflammatory cytokines, particularly IL-1β. In one studied cohort, a substantial proportion of patients with increased IL-1β levels also exhibited elevated concentrations of IL-6, TNF-α, or IL-1R, thereby delineating a coherent inflammatory profile consistent with robust activation of innate immune pathways [80]. In such cases, cytokine expression patterns suggest not only the presence of persistent inflammation but also the possible involvement of an autoinflammatory mechanism rather than a classical autoimmune response [81].

This conceptual distinction is crucial. Whereas autoimmune diseases are defined by autoantibody production and antigen-specific activation of T and B lymphocytes, autoinflammatory disorders occupy the opposite end of the immunological spectrum and are orchestrated by inflammasomes—intracellular platforms responsible for the activation and secretion of IL-1β. In the context of MD, the presence of patients with markedly elevated IL-1β levels raises the possibility that autoinflammatory mechanisms may play a more prominent role than previously assumed [80,82].

Structural or functional abnormalities of the endolymphatic sac may lead to an exaggerated immune response to specific antigens, thereby promoting the initiation and persistence of multiple inflammatory processes within the inner ear. In this context, several studies have proposed that autoinflammatory mechanisms may contribute to the molecular pathogenesis of MD, in a manner like that described in autoimmune inner ear disease (AIED), a rare condition characterized by rapidly progressive sensorineural hearing loss [8,83]. AIED and MD share substantial clinical and biological overlaps. Both conditions may present fluctuating or progressive sensorineural hearing loss, recurrent episodes of vertigo, and, in some cases, a favorable response to immunosuppressive treatment, particularly corticosteroids. These observations have led to the hypothesis that MD may encompass one or more immune-mediated endophenotypes [9].

Although the precise mechanisms underlying AIED are not yet fully elucidated, patients diagnosed with this condition frequently exhibit elevated levels of proinflammatory cytokines, including IL-1β and TNFα. These inflammatory markers suggest a direct involvement of the immune system in the progressive deterioration of inner ear structures and, at the same time, help explain the favorable response observed in many patients to corticosteroid therapy, which can modulate inflammation and suppress excessive immune reactions [65]. In this context, the link between autoimmunity and MD provides an important perspective on disease etiology and underscores the need to further investigate the role of cytokines and autoinflammatory mechanisms in the development and progression of this complex disorder. Such an approach may pave the way for more targeted and personalized therapeutic strategies for patients exhibiting pronounced autoimmune or inflammatory components [8].

From a therapeutic standpoint, several approaches have been proposed for forms of MD associated with autoimmune or autoinflammatory mechanisms, including IL-1β and IL-6 inhibitors. However, the efficacy of these treatments has not yet been systematically evaluated, and controlled clinical trials are required, particularly in patients with elevated levels of proinflammatory cytokines. This represents a promising direction for future research, focusing on targeted therapies guided by the individual immunological profile of patients with MD [58]. The demonstrated effectiveness of intratympanic corticosteroid therapy (one of the few interventions capable of stabilizing symptoms in refractory forms of the disease) further supports this perspective and suggests a major role for immuno-inflammatory processes in disease dynamics [80].

A significant increase in antiphospholipid antibody levels has been observed in patients with Ménière’s disease, suggesting a potential role in the pathogenesis of inner ear dysfunction. Mechanistically, these antibodies may impair labyrinthine microcirculation by activating endothelial cells and inducing oxidative stress, thereby promoting microthrombus formation and local ischemia. This process may contribute to dysfunction of cochleovestibular structures. From a therapeutic perspective, antiphospholipid syndrome is managed by preventing thromboembolic events, primarily using anticoagulants (of which warfarin has demonstrated clear efficacy) as well as through immunotherapy. Glucocorticoids may provide additional benefit due to their anti-inflammatory and endothelial-protective effects. Although these findings support a possible involvement of antiphospholipid antibodies in certain forms of inner ear dysfunction, further studies are required to clarify the underlying mechanisms and to evaluate the effectiveness of these therapeutic strategies in Ménière’s disease [58,64,66].

An essential aspect that requires clearer delineation is the status of autoantibodies in MD. At present, no specific autoantibody with a well-defined antigenic target in the inner ear has been identified can be consistently correlated with the onset or progression of MD. Unlike other autoimmune diseases, where the relationship between autoantibodies and tissue damage is directly demonstrable, in MD most of the described autoantibodies appear to be nonspecific or transient. This observation suggests that their presence may reflect secondary immune activation or a systemic inflammatory response rather than a primary autoimmune mechanism directed against cochleovestibular structures. Consequently, the role of autoantibodies in MD should be interpreted with caution as a possible marker of immune dysregulation rather than a well-established causal factor [58,70,80].

Another important issue is the positioning of MD within the spectrum of immune-mediated disorders. Current evidence does not convincingly support the classification of MD as a classical autoimmune disease, in the absence of defining criteria such as the identification of a specific autoantigen, demonstration of passive disease transfer, or the existence of robust experimental models mediated by autoantibodies. Instead, the immunological profile described in several studies (characterized by increased proinflammatory cytokines, activation of innate immunity, and variability in inflammatory responses) suggests a greater involvement of autoinflammatory mechanisms or chronic low-grade inflammation. This distinction is not merely conceptual but has direct implications for data interpretation and therapeutic approaches. Thus, MD may represent a heterogeneous spectrum of immunological endophenotypes, in which autoimmunity plays a role only in a subset of patients, while in others nonspecific inflammatory mechanisms or dysregulation of innate immunity predominate [8,60,64,70].

In contrast to other autoimmune conditions, where autoantibodies are directly involved in tissue injury and can be used for both diagnosis and patient stratification, in MD the evidence remains indirect and inconclusive. Existing studies are limited by small sample sizes, lack of replication, and methodological heterogeneity, and the temporal relationship between the appearance of autoantibodies and the onset of symptoms is not clearly established. Nevertheless, the existence of patient subgroups in which autoimmune mechanisms are more prominent cannot be excluded, which justifies continued interest in identifying and characterizing potential autoantibodies of clinical relevance.

Overall, current evidence supports the notion that autoimmunity may contribute to MD through multiple mechanisms, including the formation of circulating immune complexes, aberrant activation of T lymphocytes, dysregulation of immune clearance processes, and persistent inflammation within the inner ear. Nevertheless, the heterogeneity of findings indicates that autoimmunity is unlikely to represent a single, unifying explanation, but rather one of several major factors that, in interaction with genetic predisposition and environmental influences, contribute to disease pathogenesis. Future multicenter studies with large cohorts and integrated genetic and immunological analyses are required to validate these hypotheses and to assess the therapeutic potential of immunomodulatory strategies in the management of MD.

### 5.4. Allergies and Meniere’s Disease

Allergies are considered a potential triggering factor that, in the presence of intrinsic susceptibility (whether genetic, immunological, or structural) may promote the development of EH. This hypothesis is supported by the observation that not all individuals with allergies develop MD; however, the prevalence of allergic phenomena is significantly higher among patients diagnosed with this disorder [58,84].

Mechanisms such as type I hypersensitivity reactions, specific genetic features of the immune system, or the presence of circulating immune complexes are regarded as potential pathogenic pathways. Derebery suggested that the endolymphatic sac might play an active role in antigen processing, thereby initiating a localized immune response [84,85]. Both his studies and subsequent observations have demonstrated an increased prevalence of allergies in patients with MD compared to the general population [86]. Similar findings were reported by Pan Tao, who, in a study of 35 patients, identified higher rates of allergic sensitization, suggesting that IgE-mediated immune reactions may contribute to the development of MD [87,88].

**Figure 3:**
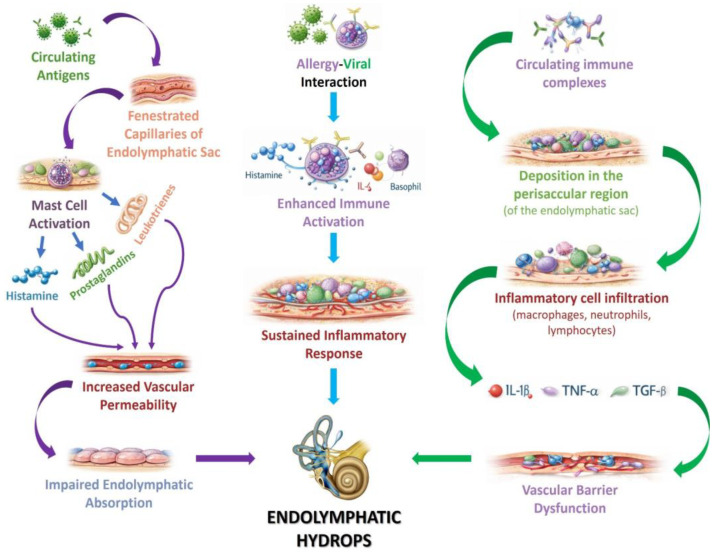
The impact of allergic components on endolymphatic homeostasis.

Derebery and Berliner proposed three main pathogenic mechanisms explaining how allergic reactions can induce local inflammation and disrupt endolymphatic homeostasis [84,87]. The first mechanism involves the penetration of antigens through the fenestrated vessels of the endolymphatic sac, a structure known for its high permeability and immunologically active role. Contact of antigens with the perisacular connective tissue can stimulate mast cell degranulation, releasing histamine and other proinflammatory mediators, which increases vascular permeability, causes tissue edema, and impairs endolymph absorption mechanisms [87,89]. The second mechanism involves the formation and deposition of circulating immune complexes, frequently generated in response to food or inhalant allergens. These complexes may accumulate along the fenestrated vessels of the endolymphatic sac, inducing chronic local inflammation and alterations of the vascular barrier. Increased permeability and the resulting inflammation can disrupt the balance between endolymph production and resorption, promoting the development of EH. The third proposed mechanism concerns a complex interaction between viral infections and the allergic response. Viruses may amplify allergic reactions by increasing histamine release, damaging the epithelium of the endolymphatic sac, and stimulating T lymphocyte migration to this structure, thereby exacerbating local inflammation [90,91]. The mechanisms of action are illustrated in Figure 3.

Pan et al. reported a significantly higher prevalence of allergies in patients with MD compared to healthy controls [88]. Furthermore, patients with a history of MD frequently exhibit elevated IgE levels (a classical marker of immediate hypersensitivity) with increased values observed in 43.3% of patients versus 19.5% in control groups [89]. These findings suggest the presence of an active immunological substrate in a subset of patients with MD. Consistently, Savastano et al. identified elevated levels of circulating immune complexes, proinflammatory cytokines, and autoantibodies in patients with MD, reinforcing the hypothesis of systemic immune and inflammatory involvement in disease pathogenesis [92].

First mechanism: Circulating antigens in the body reach the fenestrated capillaries of the endolymphatic sac, where they trigger mast cell activation, resulting in the release of histamine, prostaglandins, and leukotrienes. This leads to increased vascular permeability, affecting endolymph absorption. Second mechanism: Viruses can amplify allergic reactions by increasing histamine release, damaging the epithelium of the endolymphatic sac, and stimulating T lymphocyte migration to this structure, thereby intensifying local inflammation. This results in excessive histamine, basophils, and interleukins producing a sustained inflammatory response that over time disrupts endolymph homeostasis, leading to hydrops and the characteristic symptoms of the disease. Third mechanism: Circulating immune complexes deposit in the perisacular region, inducing a strong inflammatory process that damages the vascular barrier, resulting in the development of endolymphatic hydrops.

Population-based studies provide an opportunity to explore the connections between MD and various comorbid conditions. Analysis of these studies reveals significant differences across populations: in East Asia, MD correlates with allergic respiratory diseases (chronic obstructive pulmonary disease (COPD), fibromyalgia, and vitiligo), whereas in the European population from the UK, associations have been identified with rheumatoid arthritis and psoriasis. Remarkably, the relationship with allergic conditions was replicated in three independent studies from East Asia [93,94,95], while this association was not observed in European cohorts. Conversely, rheumatoid arthritis and psoriasis did not show links with MD in East Asia [96]. This population-specific variability indicates distinct interactions between local environmental factors and immunological susceptibility, suggesting that allergic inflammation is more prevalent in East Asia, whereas European populations may have an increased predisposition toward autoimmunity [57,58].

Most of the available population studies originate from Asia, whereas European data are limited to a single large-scale study based on the UK Biobank [3]. This uneven distribution may influence the interpretation of associations with MD, as clinical phenotypes and comorbidities vary between populations, and the immune response may be differently modulated by the interaction between genetics and environment. Differences in the prevalence of allergic and autoimmune diseases between East Asia and Europe have been particularly evident regarding allergic conditions in the meta-analysis conducted by Lopez et al. [58]. Nevertheless, additional population-based studies in various European regions are required to accurately evaluate the role of allergies in MD, considering climatic variations and differing allergen exposures across the Mediterranean, Central Europe, and Scandinavian regions [58,88,93].

Special attention has been directed toward food allergies, particularly those associated with wheat and gluten. Derebery identified wheat, particularly gliadin, as the most common food allergen in patients with MD [97]. Gliadin is the primary determinant of IgE-mediated hypersensitivity in wheat allergy and can trigger both immediate reactions and delayed inflammatory responses [98,99]. These findings have paved the way for more detailed investigations into the role of gluten in MD. Di Berardino et al. assessed IgE hypersensitivity to gliadin in pharmacologically untreated patients with MD, comparing them with healthy subjects and patients with grass pollen–induced allergic rhino conjunctivitis. Skin prick testing revealed positive reactions to gliadin in a significant subset of patients with MD, in both the immediate and delayed phases, whereas the control groups showed negative results. These findings suggest the existence of MD-specific immunological mechanisms that are distinct from those observed in other common allergic conditions [99,100].

In a subsequent study, increased immunological reactivity to gluten biomarkers was demonstrated in patients with MD. Serological analysis revealed elevated levels of anti-wheat IgA, anti-cerebellar peptide IgA, and anti–glutamate decarboxylase (GAD) IgM, compared with healthy controls. Notably, these immunological alterations were confirmed in a subgroup of patients who exhibited clinical improvement following the adoption of a gluten-free diet, suggesting a direct link between gluten-induced immune activation and MD symptomatology [88,99]. Although data on allergy-targeted therapeutic interventions in MD remain limited, the available studies indicate potential clinical benefits. Patients undergoing specific immunotherapy or elimination diets for food allergens reported reductions in the frequency and severity of vertigo attacks, as well as improvements in associated symptoms, including tinnitus and vestibular instability. In this context, systematic assessment of allergy history and the performance of allergological testing may represent an important step toward a personalized management approach for patients with MD, particularly those with refractory forms or evident immunological comorbidities [99].

While a direct causal relationship between allergy and MD has not yet been conclusively established, the available evidence supports the notion that allergies, whether food-related or seasonal, may act as triggers or exacerbating factors for symptoms in a susceptible patient subgroup. Accordingly, the identification and appropriate management of allergies could have a beneficial clinical impact by reducing the frequency and severity of MD symptoms [9]. Therefore, allergological evaluation should be considered as part of a multidisciplinary approach to patients with MD.

The allergic hypothesis, although supported by a higher prevalence of allergic phenomena in patients with MD, is affected by similar methodological limitations. Most studies do not adequately control confounding factors such as atopic comorbidities or environmental exposure, and the temporal relationship between allergen exposure and symptom onset is not always clearly defined. In addition, the lack of controlled interventional studies consistently demonstrating the benefit of antiallergic therapy limits the strength of this hypothesis, suggesting that allergies may act more as triggering or aggravating factors rather than primary determinants of the disease.

### 5.5. Viral Infections

Another major etiological hypothesis for MD is viral involvement. This hypothesis is considered due to the high prevalence of latent viral infections in the general population and the ability of certain neurotropic viruses to induce recurrent inflammation in neural structures However, direct evidence is difficult to obtain, primarily because the membranous labyrinth is largely inaccessible for routine microbiological investigations [1,6].

The viral hypothesis in MD is also supported by the fluctuating and recurrent nature of symptoms, the initial unilateral onset followed by progressive disease, and clinical similarities with other vestibulo-cochlear disorders of viral etiology. In most cases, viral infections are not considered sole or direct causes of MD but rather triggering or modulating factors, capable of initiating or sustaining chronic inflammatory processes within the inner ear. These processes may interact with genetic predisposition and other environmental factors, contributing to the development of progressive vestibulo-cochlear dysfunction [101,102]. Among the viruses investigated, those most frequently associated with MD are herpesviruses (particularly HSV-1), varicella-zoster virus (VZV), cytomegalovirus (CMV), and, more rarely, Epstein–Barr virus (EBV). These viruses are neurotropic and capable of establishing long-term latent infections in sensory ganglia, including the spiral and vestibular ganglia. The characteristic viral latency and periodic reactivation provide a biologically plausible framework for the recurrent nature of vertigo attacks in MD [1,2,103].

**Figure 4:**
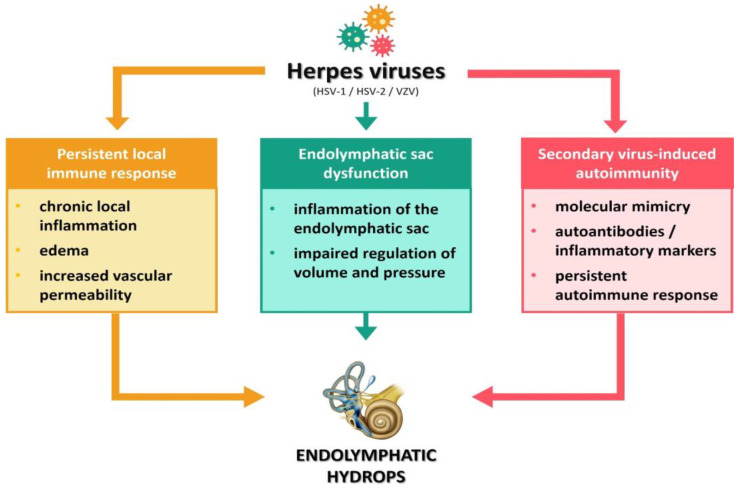
The mechanism of action of Herpesviruses in Ménière’s disease.

Viral infections, particularly herpesviruses, have been proposed as factors involved in triggering or maintaining inner ear inflammation. The recurrent symptoms, alternating periods of remission and exacerbation, aggravation under stress, and tendency toward progressive improvement are features shared by MD and herpesvirus reactivation, supporting the plausibility of this theory [1,6]. Viruses may induce a persistent local immune response, promote cross-reactive autoimmune reactions, and alter the function of the endolymphatic sac (Figure 4).

A central mechanism proposed is latent viral persistence within the vestibular nerve or the sensory structures of the inner ear. Periodic viral reactivation may induce local inflammation, neural edema, and altered sensory signal transmission. Over time, these repeated episodes can result in cumulative damage to the sensory epithelium and structures involved in endolymph volume regulation, promoting the development of EH. Histopathological studies have identified viral D NA within inner ear structures and demonstrated inflammatory and degenerative changes consistent with chronic viral insults. Viral infections may impact endolymphatic sac function by inducing a local inflammatory response characterized by lymphocytic infiltration, release of proinflammatory cytokines, and increased vascular permeability [101,104,105].

Another important mechanism is the activation of a secondary autoimmune response induced by viral infection. Through molecular mimicry, certain viral antigens may trigger the production of autoantibodies that cross-react with structures of the inner ear. This hypothesis is supported by the identification of autoantibodies and inflammatory markers in a subset of patients with MD, suggesting that viruses can initiate a persistent autoimmune process even after the resolution of active infection. Not all individuals exposed to viruses develop MD, indicating the need for a susceptible genetic background. Variability in the antiviral immune response, differences in the expression of genes involved in inflammation, and the homeostasis of the inner ear may explain why viral infections trigger MD only in certain individuals [3,9,69].

The results of studies have been contradictory. Welling et al. did not identify viral DNA in the vestibular ganglia [106], whereas Vrabec et al., using more sensitive PCR techniques, reported the presence of viral DNA in both patients with MD and control subjects [107]. Gartner et al. did not detect genomic HSV or VZV DNA in patients with well-defined MD, concluding that reactivation of these viruses does not play a major role in the pathogenesis of the disease [103].

Host Cell Factor C1 (HCFC1) is an essential cellular factor known for its ability to interact directly with certain HSV proteins, facilitating critical steps of viral replication within host neurons. This interaction suggests that HCFC1 may play a central role in the host response to herpetic infections in the nervous system [2]. The study conducted by Vrabec et al. provided further evidence for the possible involvement of viral mechanisms in the pathogenesis of MD. A significant increase in the frequency of the primary allele of SNPs within the HCFC1 gene was observed among patients diagnosed with MD, whereas the secondary allele appeared to confer a protective effect, being more common in healthy individuals [105].

This genetic association strengthens the hypothesis that reactivation or persistence of HSV infection could represent a potential etiological factor in triggering or exacerbating the disease. The presence of HCFC1 gene variants that favor viral replication in neuronal cells may create a susceptible environment for inflammation, immune dysregulation, or endolymphatic dysfunction. Therefore, the involvement of HCFC1 not only supports a potential viral model of MD but also opens new avenues for research on the interaction between genetic susceptibility and latent infections [2,105].

Most of the studies we identified on this topic are outdated, and another issue is their low number, which may lead to premature conclusions or results that are not fully consistent with reality. Current studies on this subject are needed to establish clearer conclusions. Although there is biological and clinical evidence supporting the role of viral infections in MD, the results are not consistent across studies, and the detection of viral DNA does not necessarily imply a direct causal relationship. These limitations suggest that viral infections are more likely contributory factors rather than sole determinants of the disease. Nevertheless, understanding this mechanism has important clinical implications, including for patient stratification and the exploration of antiviral or immunomodulatory therapies in selected patient subgroups.

Data regarding the role of viral infections are also contradictory. Although some studies have identified viral DNA in vestibular structures, these findings are not consistent and have also been replicated in healthy subjects. This lack of specificity raises important questions regarding the clinical significance of these findings and suggests that viral presence may reflect a common latency rather than a direct pathogenic mechanism. In the absence of longitudinal evidence correlating viral reactivation with clinical episodes, the viral hypothesis remains biologically plausible but insufficiently demonstrated.

### 5.6. Age

Age represents a significant non-genetic factor in MD, exerting a direct impact on both the epidemiology and progression of the disease. Epidemiological analyses from various regions indicate that MD is predominantly an adult disorder, rarely observed during childhood or adolescence. This suggests that the biological mechanisms related to vestibular system maturation and alterations in inner ear fluid homeostasis may accumulate over a lifetime before manifesting clinically. Most patients with MD experience symptom onset between 40 and 60 years of age, the period during which prevalence is highest, and diagnosis is most frequently established. In multiple epidemiological studies, the mean age at first vertigo episode or at clinical diagnosis is approximately 42–50 years, both in sporadic and familial cohorts, with slight variations among subgroups and populations [3,8,87,108].

In the younger age category, the disease is uncommon: less than 3% of cases occur in individuals under 18 years old, and presentation during childhood is exceptional. This pattern supports the notion that the pathogenic mechanisms leading to EH require a certain “biological time” or cumulative exposure to factors before being triggered [1,109,110]. At the opposite end of the spectrum, MD can also appear in advanced age, although these cases are less frequent in most published series. Some cohorts indicate that approximately 10% of patients may develop symptoms after 65 years, either as a late-onset disease or as a reactivation of pre-existing MD. These cases suggest that aging of the vestibular system and reduced physiological reserves may act as factors favoring the clinical manifestation of the disease in elderly individuals [87,108].

The increased prevalence of MD in older populations is a well-documented phenomenon, reported in multiple epidemiological studies [87,108,111,112]. Although some European data describe a decrease in prevalence after the age of 70 [111], observations from the study by Simo et al. indicate a continuous increase up to approximately 90 years of age, followed only subsequently by a decline [113]. This discrepancy among reported findings may be explained by factors such as improved diagnostic access, increased life expectancy, and a higher likelihood of elderly patients being included in hospital databases. Additionally, the decline reported in some European studies after 70 years may be influenced by increased mortality in this age group, which reduces the number of individuals available for diagnosis, rather than reflecting a true decrease in disease risk [113].

From a pathophysiological perspective, MD is described as a degenerative disorder resulting from dysfunction of endolymphatic homeostasis mechanisms, characterized by alterations in ionic composition and disruption of the balance between endolymph production and absorption. This view is supported by the concept that the pathological process evolves slowly, driven by a combination of genetic, vascular, autoimmune, inflammatory, or metabolic factors that act cumulatively over the course of life [114]. Findings from the study by Simo et al. demonstrate a progressive increase in disease prevalence between 20 and 90 years of age, fully consistent with this pathophysiological model. These findings suggest insidious progression, potentially accelerated by cumulative exposures or by gradual loss of compensatory mechanisms within the inner ear. Moreover, this continuous progression into older age raises the hypothesis that MD may, at least in part, reflect the cumulative effects of aging on the endolymphatic system concept increasingly discussed in recent literature [113].

Epidemiological data suggest that the mean age of onset may differ slightly among populations from different geographic regions or with distinct demographic characteristics. For example, some studies suggest a slightly higher mean age of onset in Western European cohorts compared with data from Asia, where the overall disease prevalence is lower [115]. In Taiwan, the age of onset has been observed to shift toward later decades of life in recent periods, reflecting both population aging and potential influences of socioeconomic and environmental factors. Additionally, age-related variations in disease prevalence are often correlated with comorbidities that increase with advancing age, such as vascular or metabolic disorders, which may influence susceptibility to endolymphatic dysfunction. Thus, age is not merely a demographic marker, but rather a factor reflecting the cumulative evolution of biological and environmental interactions that can drive the onset and progression of MD [3,111,115,116].

### 5.7. Sex

The distribution of MD according to sex has traditionally been considered relatively balanced, although numerous studies have reported a slight female predominance [113,117]. Some older epidemiological analyses even suggested the possibility of a decreasing proportion of affected women over time [118]; however, such observations should be interpreted with caution, given the variability in diagnostic criteria and methodological differences across studies. Findings from the study by Simo et al. contradict this downward trend and indicate a clear female predominance, with an odds ratio (OR) of 1.51, which is consistent with several reports confirming the same epidemiological orientation [113,117]. This discrepancy between earlier and more recent studies may reflect changes in healthcare-seeking behavior, sex-related differences in sensitivity to vestibular symptoms, and potential hormonal influences on endolymphatic homeostasis, an area that remains insufficiently explored [113,115].

The reason why women are more frequently affected is not fully elucidated, but several biological hypotheses may contribute to this observation. One potential factor is the role of sex hormones, particularly estrogen, which can influence inner ear fluid homeostasis. Estrogen and progesterone may modulate fluid balance, vascular permeability, and immune responses—all mechanisms implicated in the pathogenesis of MD. Although direct evidence remains limited, these hormonal influences may partially explain why women are more susceptible to symptom manifestation, particularly during periods of hormonal fluctuation such as the menstrual cycle, pregnancy, or menopause. Additionally, associated conditions such as autoimmune disorders, allergies, or migraine are more common in women and may interact with MD risk, amplifying female susceptibility in certain clinical contexts [87,113,119].

It is important to note that evidence regarding sex as a risk factor is not universally conclusive. A recent meta-analysis identified a trend toward a higher association in women; however, statistical significance was not consistently reached across all analyzed datasets, suggesting that claims regarding sex as a prognostic factor remain tentative and may vary depending on study methodology and populations investigated. This reflects the clinical and epidemiological heterogeneity of MD, as well as the complex influence of genetic and environmental factors interacting with biological sex [87].

### 5.8. Race and Ethnicity

The distribution of MD varies significantly across populations, suggesting that race and ethnicity act as important non-genetic factors, yet are closely interconnected with the specific genetic background of each population. MD is most frequently reported in European and North American populations of Caucasian descent. Epidemiological studies from Europe and the USA indicate an estimated prevalence ranging from 50 to 200 cases per 100,000 individuals, with a significantly higher incidence compared to other ethnic groups. These populations have also yielded most identified genetic associations, including HLA variants, as well as genes involved in inflammation, ion homeostasis, and immune response. Higher prevalence may reflect both genuine biological susceptibility and more frequent diagnosis due to better access to advanced vestibular and imaging assessments [3,48,108,117].

In the United States, the distribution of MD across different ethnic groups remains an insufficiently clarified area of study. The prevalence among African American populations has been reported, depending on the study, as either lower [120] or like that observed in Caucasians [121], suggesting the presence of significant confounding factors, such as access to healthcare services, patterns of medical presentation, or cultural differences in reporting vestibular symptoms. Other ethnic groups, including Hispanic, Asian, and Native American populations, have shown even lower rates of the disease [116,117,121,122], raising the possibility of a protective genetic background or, alternatively, underdiagnosis due to linguistic, socio-economic, or access barriers. Current study results align with this trend and confirm lower prevalence among African Americans, extending this epidemiological pattern to other ethnic categories as well [108,113].

Estimates of MD prevalence in Japan have fluctuated considerably over recent decades, reflecting methodological diversity among studies and inherent difficulties in clearly defining cases. Published data from the past 35 years indicate values ranging from 3.5 to 73 per 100,000 individuals, variations that can be explained by differences in sampling, diagnostic criteria, and data collection tools used in various epidemiological surveys [113]. More recent estimates have stabilized within a narrower range, between 20 and 35 per 100,000 [112,116], suggesting improved standardization of research methods and, likely, greater consistency in the application of diagnostic criteria. Compared with the USA and Europe, the reported prevalence in Japan remains lower, raising questions regarding genetic, cultural, or environmental influences on the clinical expression of the disease. Interestingly, many genetic associations identified in European populations, particularly those within the HLA region, have not been replicated in Asian cohorts. This discrepancy suggests that the population-specific structure of HLA haplotypes and differences in linkage disequilibrium may modify the impact of genetic variants on disease risk [73,113,123].

In Sub-Saharan African regions, however, the epidemiological picture remains considerably less clear. Some studies have reported higher prevalences of MD compared to Europe or the USA [124], whereas others have described substantially lower values [125]. This inconsistency likely reflects both the heterogeneity of research methodologies employed and deficiencies in medical infrastructure and data collection. In the absence of uniformly accepted diagnostic criteria and robust reporting systems, direct comparisons are problematic. Beyond these limitations, recent genetic research suggests that racial and ethnic differences may have a significant biological component, influencing individual susceptibility to MD [113,117]. This line of investigation may help clarify in the future the extent to which the global distribution of the disease reflects genuine differences in biological predisposition or, conversely, artifacts of epidemiological methods. Race and ethnicity should not be regarded as direct determinants of MD, but rather as markers of a broader biological and socio-genetic context.

### 5.9. Environmental and Socio-Economic Factors

The potential role of environmental factors in the etiology of MD has been discussed for several decades; however, initial hypotheses in this regard were met with considerable skepticism. In the absence of robust evidence, the scientific community initially viewed with caution the idea that geographic, climatic, or socio-environmental variables could modulate the risk of disease onset. Nevertheless, a study conducted in Nigeria suggested that differential access to medical facilities could contribute to the observed variations in prevalence [124]. Although these findings were relevant within the African context, their transferability to populations in the United States or Europe remained uncertain, due to both systemic differences in medical infrastructures and socio-economic variability [113]. A subsequent large-scale study confirmed this caution, indicating that data from Nigeria cannot be directly extrapolated to the American population [118].

Reports of higher MD prevalence among medical professionals add another dimension to the discussion of environmental or occupational influences. The hypothesis of increased vulnerability in these groups may reflect a combination of occupational stress, exposure to specific acoustic conditions, better access to diagnostic facilities, or heightened symptom awareness. The study by Simo et al. highlights increased prevalence among high-income populations, which aligns with this perspective. Notably, the effect of income on prevalence appears to be independent of race, suggesting that socio-economic status influences the disease through distinct mechanisms, likely related to access to specialized medical evaluations, health behaviors, or occupational factors [113].

Analysis of geographic distribution reveals another important trend: MD prevalence is higher in rural environments compared to urban areas. As population density increases, prevalence decreases, suggesting a potential contribution of variables such as exposure to specific dietary patterns, environmental conditions characteristic of less industrialized areas (e.g., humidity, allergens, or exposure to agricultural toxins), as well as differences in health-seeking behaviors. Additionally, factors such as communication with physicians, health literacy, healthcare system structure, and even cultural variations in reporting vestibular symptoms may contribute to these differences [113].

Overall, these observations suggest that the prevalence of MD cannot be explained solely by biological predisposition, but rather reflects a complex interaction between environmental factors, socioeconomic status, patterns of access to healthcare services, and occupational determinants. However, the existing evidence is insufficient to establish definitive causal relationships, and inconsistencies across different geographic regions and professional groups highlight the need for more rigorous studies. Therefore, further research is required to precisely delineate the influence of extrinsic factors on the epidemiology of MD.

### 5.10. Diet

In patients with MD, nutrition plays a crucial adjuvant role in symptom control. The primary goal of dietary management is to maintain stable fluid balance and avoid hormonal and metabolic fluctuations that may influence endolymphatic volume in the inner ear. In MD, there are no “forbidden foods” in an absolute sense; however, certain foods and beverages are strongly discouraged, as they may promote fluid retention, affect endolymphatic pressure, or trigger vertigo attacks. Recommendations are based on clinical observations and known pathophysiological mechanisms of the disease. There are several dietary principles that patients with MD should be guided to follow.

Sodium restriction is one of the oldest and most frequently used non-pharmacological interventions in the management of MD. Although there are currently no specific guidelines defining an optimal sodium threshold exclusively for MD patients, general dietary recommendations advise limiting daily sodium intake to less than 2300 mg for individuals aged 14 years and older. This recommendation is frequently extrapolated to MD patients, based on clinical observations that high salt consumption may precipitate or exacerbate acute episodes of vertigo, tinnitus, and aural fullness [99,126].

However, the relationship between sodium intake and the pathophysiology of MD has proven more complex than the initial hypothesis of a direct correlation between serum sodium levels and EH formation. Experimental and clinical studies, both in animal models and human subjects, have shown that low-sodium diets do not induce significant changes in plasma sodium concentration. Moreover, serum sodium levels typically remain within the normal range, even among patients with histopathological confirmed EH. These findings suggest that the mechanisms by which sodium restriction influences MD progression are not directly mediated by plasma sodium variations but involve endocrine processes and local regulatory mechanisms within the inner ear [99,127].

A central proposed pathophysiological mechanism is related to the activation of the renin–angiotensin–aldosterone system under conditions of low salt intake. Sodium restriction is associated with a compensatory increase in plasma aldosterone, a mineralocorticoid hormone that plays a key role in hydro-electrolytic balance. Studies have demonstrated that aldosterone stimulates endolymph absorption in the inner ear, contributing to the maintenance of endolymphatic volume. Mineralocorticoid receptors have been identified in the endolymphatic sac, suggesting a direct local sensitivity to this hormone [128,129].

In addition, multiple ionic transport systems involved in sodium homeostasis are expressed at the epithelium of the endolymphatic sac, including epithelial Na^+^ channels, Na^+^/K^+^-ATPase, and the thiazide-sensitive Na^+^-Cl^−^ cotransporter, all of which are regulated, at least in part, by aldosterone [130,131]. The endolymphatic sac exhibits increased Na^+^ permeability and high Na^+^/K^+^-ATPase activity, features that support its capacity to absorb excess endolymph. In this context, the rise in aldosterone levels induced by a low-sodium diet could promote endolymph absorption, thereby reducing EH [32,99].

The clinical efficacy of sodium restriction was evaluated in a longitudinal study in which 13 patients with MD followed a low-sodium diet (~2 g Na/day) over a two-year period. In this protocol, 24 h urine samples were collected at the start of the diet and subsequently at 2, 4, 6, and 8 weeks, as well as at 6, 12, 18, and 24 months. Biological assessment included measurements of plasma osmolality, aldosterone, cortisol, antidiuretic hormone, and serum concentrations of Na^+^, K^+^, and Cl^−^. The results indicated that the low-sodium diet was associated with clinical symptom improvement and increased ionic transport and plasma aldosterone levels, supporting the hypothesis of enhanced endolymph absorption at the endolymphatic sac. Based on these data, the authors recommended the systematic exclusion of high-sodium foods as an integral part of dietary management in MD patients [99,132].

In addition to added salt, monosodium glutamate (MSG) may play a relevant role. MSG is a common food additive in processed foods, canned goods, instant soups, sauces, salad dressings, and meat products, particularly in Asian cuisine. Given its high sodium content and potential to increase total sodium load, reducing MSG intake has been proposed as an adjunct to sodium restriction and may help prevent or alleviate MD symptoms [99]. Overall, available evidence supports a low-sodium diet as an adjuvant strategy in MD management, not by directly altering plasma sodium, but by activating endocrine and ionic transport mechanisms that favor regulation of endolymphatic volume. However, larger controlled studies are needed to clarify clinical benefits, define optimal sodium restriction thresholds, and identify patient subgroups most likely to respond to this intervention.

A balanced glycemic diet is also recommended, as rapid fluctuations in blood glucose and insulin secretion can influence inner ear microcirculatory blood flow. Postprandial hyperglycemia and episodes of reactive hypoglycemia may promote autonomic instability, increased oxidative stress, and changes in vascular permeability, all of which are associated with exacerbation of vestibular symptoms. Maintaining a stable glycemic profile contributes to reducing the frequency of vertigo episodes and associated autonomic symptoms. Regular meals at approximately equal intervals are recommended to prevent abrupt metabolic and hormonal fluctuations that may influence endolymph homeostasis. Prolonged fasting or irregular meals can induce hypoglycemia, activate the stress axis, and alter cortisol and catecholamine secretion, factors that may trigger or worsen vertigo attacks. A consistent eating schedule supports metabolic stability and reduces physiological stress on the vestibular system [132,133].

Adequate hydration, distributed evenly throughout the day, plays an important role in maintaining osmotic balance and preventing compensatory fluid retention. Insufficient fluid intake can lead to increased plasma sodium concentrations and activation of hormonal water-conservation mechanisms, whereas the sudden ingestion of large volumes of fluids may temporarily disrupt hydro-electrolytic balance. Consistent and uniform hydration helps stabilize endolymphatic volume and reduces the risk of symptomatic fluctuations [32,130,131].

Caffeine is a plant-derived alkaloid widely present in the human diet, commonly found in coffee, tea, cocoa, cola beverages, energy drinks, and chocolate-containing products. Chemically, caffeine belongs to the methylxanthine class, compounds known for their stimulatory effects on the central nervous system, cardiovascular system, and water metabolism. In the context of MD, caffeine has been considered a potential aggravating factor. One proposed mechanism relates to its vasoconstrictive effect, which may reduce blood flow in the microcirculation of the inner ear. Given that patients with EH already exhibit impaired vascular autoregulation, this additional reduction in perfusion could limit the labyrinthine structures’ ability to adapt to fluctuations in endolymph pressure and volume. Through these mechanisms, high caffeine intake may contribute to the triggering or exacerbation of vertigo episodes and may represent a shared susceptibility factor for both migraine and MD [55,99].

The clinical relevance of these observations is supported by observational data. In a study comparing total caffeine intake from coffee, tea, cola beverages, energy drinks, and chocolate-containing foods, patients with MD had significantly higher caffeine consumption compared with both patients with dizziness from other causes and healthy control subjects. These findings suggest a possible association between increased caffeine intake and susceptibility to MD, although a causal relationship cannot be definitively established [99,134].

Alcohol consumption, on the other hand, is well known for its systemic adverse effects and is associated with multiple chronic pathologies, including cardiovascular disease, liver disease, and neoplasms. Regarding the inner ear, alcohol has been proposed as a potential factor involved in the onset or exacerbation of labyrinthine dysfunction. Several studies have reported harmful effects of alcohol on cochlea and vestibular labyrinth, although experimental evidence remains heterogeneous. In the absence of definitive conclusions, the general clinical recommendation is to avoid or limit alcohol consumption in patients with inner ear disorders [99,135].

Both caffeine and alcohol exert significant effects on fluid distribution within the body. Excessive consumption of these substances can induce rapid alterations in hydro-electrolytic balance, directly impacting the electrochemical stability of inner ear fluids [114]. Caffeine is rapidly distributed across the body’s fluid compartments, primarily due to its sympathomimetic and diuretic effects mediated by methylxanthines. These properties may lead to fluctuations in endolymphatic volume and changes in intracochlear pressure, promoting vestibular instability [99].

The analysis of alcohol’s impact on MD must consider two main mechanisms, both related to the regulation of body fluids. First, alcoholic beverages contribute to total fluid intake, which can influence overall water balance and must be incorporated into the assessment of daily hydration. Second, alcohol inhibits hypothalamic secretion of vasopressin, a hormone essential for renal water reabsorption. Reduced vasopressin levels lead to increased diuresis, with secondary effects on endolymphatic volume and pressure [99]. In addition, alcohol may induce vasoconstriction and reduce inner ear blood flow, an effect that can exacerbate tissue hypoxia and worsen vestibular and auditory symptoms in patients with MD. Although literature specifically examining the interaction between caffeine, alcohol, and MD is relatively limited, most clinical studies and therapeutic guidelines include restriction of these substances as part of overall dietary modifications [136,137].

Adherence to these dietary principles represents not merely a general nutritional measure but a complementary therapeutic strategy integrated into the long-term management of MD. By influencing hydro-electrolytic, metabolic, and neuroendocrine mechanisms, diet can significantly contribute to symptom control and improve patients’ quality of life. Patients with MD should adopt a low-sodium, balanced, anti-inflammatory diet emphasizing fresh and minimally processed foods. Nutritional recommendations for MD are summarized in Table 2, based on the analysis of studies mentioned in the text. While diet does not cure the disease, it can reduce the frequency and severity of vertigo attacks, thereby substantially enhancing quality of life.

Regarding dietary interventions, although sodium restriction is widely recommended, the evidence supporting its clinical effectiveness is limited and sometimes contradictory. Many studies do not include appropriate control groups or fail to standardize sodium intake and hydration, making the interpretation of results difficult. In addition, the observed beneficial effects may be influenced by concomitant factors such as lifestyle changes or increased treatment adherence. These limitations highlight the need for randomized controlled trials to better define the role of dietary interventions and to identify patient subgroups that may benefit the most.

### 5.11. Sleep Disorders

The relationship between MD and sleep disorders is bidirectional and multifactorial, supported by clinical, pathophysiological, and observational data. Although no single mechanism is universally accepted, several explanatory pathways can clarify this association. First, the hallmark symptoms of MD directly influence sleep architecture. Episodes of vertigo, persistent tinnitus, and a sensation of aural fullness can cause difficulty initiating and maintaining sleep, frequent awakenings, and fragmented sleep. Tinnitus is particularly relevant in this context, as the perception of internal noise intensifies during nocturnal silence, increasing cortical hyperexcitability and reducing sleep quality. Nighttime vertigo attacks can, in turn, induce anticipatory anxiety, further exacerbating insomnia [138,139].

Second, sleep deprivation and circadian rhythm disruption may act as triggers or aggravating factors for Ménière’s attacks. Lack of sleep is associated with activation of the hypothalamic–pituitary–adrenal axis and increased levels of cortisol and catecholamines, which can alter vascular regulation and fluid homeostasis in the inner ear. Another important factor is the role of the neuroendocrine system and sleep-related hormones. Vasopressin (antidiuretic hormone), which exhibits circadian variation and is influenced by sleep quality, plays a role in water balance regulation. Elevated or dysregulated vasopressin levels have been associated with increased endolymph production and EH, a central mechanism in MD pathogenesis. Thus, sleep disturbances may indirectly contribute to inner ear fluid imbalances [133,139,140].

Sleep disorders are also frequently associated with comorbidities common in MD, such as anxiety, depression, migraine, and autoimmune diseases. Insomnia and non-restorative sleep can amplify the perception of vestibular and auditory symptoms, lowering the tolerance threshold for vertigo and tinnitus. Additionally, vestibular migraine, often difficult to differentiate from MD, is strongly influenced by sleep quality, suggesting shared mechanisms of central dysfunction [87].

Anxiety represents a relevant non-genetic factor in Ménière’s disease, acting not only because of vestibular symptoms but also as an element that can facilitate and amplify clinical manifestations. Through activation of the hypothalamic–pituitary–adrenal axis and the autonomic nervous system, anxiety leads to increased levels of cortisol and catecholamines, with effects on inner ear microcirculation, vascular regulation, and endolymphatic homeostasis. Thus, a vicious cycle is established in which vertigo episodes induce anticipatory anxiety, which in turn increases susceptibility to new episodes, negatively influencing disease progression and patients’ quality of life.

Data from a large study showed that obstructive sleep apnea (OSA) is associated with an increased incidence of vertigo, supporting the potential impact of intermittent hypoxia on vestibular function [141]. Similar findings were reported by Kim et al., who observed that middle-aged women and individuals diagnosed with OSA have a higher risk of developing MD [142]. This association is physiologically plausible: the inner ear is a highly metabolic organ with low hypoxia tolerance, so repeated desaturation episodes characteristic of OSAHS can compromise cochlear blood flow and alter the permeability of the labyrinthine barrier, promoting EH development. Furthermore, the literature suggests that alterations in inner ear microcirculation and dysfunction of the blood–labyrinth barrier, through increased vascular permeability, may contribute to MD progression, mechanisms that could explain the link between OSA and MD [87].

In conclusion, the relationship between MD and sleep disorders is complex and bidirectional. The symptoms of the disease affect sleep quality, while poor sleep, in turn, can exacerbate the pathophysiological mechanisms involved in MD through neuroendocrine, vascular, and immunological dysregulation. From this perspective, the evaluation and management of sleep disorders should be considered an important component of the integrated care of patients with MD.

### 5.12. Thyroid Function

One primary mechanism is related to the role of thyroid hormones in regulating metabolism and ionic transport. Thyroxine (T4) and triiodothyronine (T3) are essential for the normal function of ion pumps and sodium–potassium channels involved in maintaining endolymph composition. In hypothyroidism, reduced cellular metabolic activity can impair Na^+^/K^+^-ATPase function and ion channel activity in the cochlea and endolymphatic sac, promoting hydro-electrolytic imbalances. These changes may contribute to excessive endolymph accumulation and the development of EH [143,144].

Hypothyroidism is frequently associated with fluid retention and disturbances in water balance. Decreased renal water clearance, increased capillary permeability, and the accumulation of mucopolysaccharides in tissues (myxedema) can promote interstitial edema. In the inner ear, these phenomena may impair endolymphatic drainage and increase endolymphatic pressure, exacerbating the vestibular and auditory symptoms characteristic of MD [145].

Clinically, several observational studies have reported a higher prevalence of thyroid dysfunction in patients with MD compared to the general population. Data from large studies suggest that hypothyroidism may act as a risk factor for disease onset [144,146]. Santosh et al. also reported that a 12-week course of thyroid hormone replacement therapy led to symptom improvement in patients with MD and coexisting thyroid dysfunction [147]. However, evidence is not uniform: other studies, such as one in which only 1 of 208 patients exhibited thyroid abnormalities [148], indicate that the relationship between thyroid function and MD is less clear-cut than initially thought.

Overall, study results converge toward a consistent association between MD and autoimmune thyroid pathology, observed in both European and Asian populations. This pattern suggests a common immunogenetic basis, possibly determined by frequent genetic variants with pleiotropic effects on multiple inflammatory pathways. One such pathway is the TWEAK/Fn14 axis, whose regulation appears influenced by an eQTL (rs4947296) associated with both bilateral forms of MD and early-onset hypothyroidism. It is likely, however, that other common variants related to MD and thyroid autoimmunity also contribute to this relationship and require identification in future studies [58,79,87].

Another major aspect is the autoimmune component. The most common cause of hypothyroidism is Hashimoto’s autoimmune thyroiditis, and MD has been associated with various autoimmune conditions. This overlap suggests the existence of a shared immune endophenotype. Chronic immune activation, autoantibody production, and proinflammatory cytokine release may affect the endolymphatic sac, which has an active immunological role. Local inflammation could impair endolymph absorption and contribute to inner ear dysfunction. Additionally, hypothyroidism is frequently associated with nonspecific neurological and sensory symptoms, such as dizziness, postural instability, hearing loss, or tinnitus. These manifestations may mimic or exacerbate the clinical picture of MD, delaying differential diagnosis and suggesting a possible interaction between thyroid dysfunction and the vestibular system [143,145,146].

Hypothyroidism may also influence microvascular circulation. Thyroid hormones play a key role in maintaining vascular tone and blood flow. In hypothyroidism, decreased cardiac output and increased peripheral vascular resistance can lead to tissue hypoperfusion. Given the inner ear’s sensitivity to variations in blood flow, such changes may impair the oxygenation and metabolic support of vestibulocochlear structures, thereby contributing to the onset or exacerbation of Ménière’s symptoms [58,143].

In conclusion, hypothyroidism can be considered both a risk factor and a modifier of MD progression through multiple mechanisms, including ionic transport dysregulation, fluid retention, autoimmune inflammation, and microvascular dysfunction. Although not a sole determinant of the disease, assessment of thyroid function is warranted in patients with MD, particularly in the presence of suggestive symptoms or other autoimmune comorbidities. Integrating endocrine management into the multidisciplinary care of MD may contribute to more effective symptom control and improved patient quality of life.

## 6. Discussion and Conclusions

Ménière’s disease is currently recognized as a multifactorial disorder in which genetic predisposition, although relevant, is not sufficient to explain the clinical onset, fluctuating course, and phenotypic heterogeneity observed among patients. Analysis of non-genetic factors highlights their essential role in modulating the pathophysiological mechanisms involved in the disease, acting both as triggers of symptoms and as determinants influencing the progression and severity of clinical manifestations [87,115].

From a pathophysiological perspective, non-genetic factors interfere with inner ear regulatory mechanisms through several converging pathways. One of the most important involves disruption of hydro-electrolytic homeostasis. Diet, hydration status, hormonal balance, and renal function directly influence the activity of ionic transport systems expressed in the endolymphatic sac, including epithelial sodium channels, Na^+^/K^+^-ATPase, and Na^+^–Cl^−^ cotransporters [32,34,149]. Changes induced by sodium intake, fluctuations in aldosterone or vasopressin levels, and endocrine imbalances can impair endolymph absorption, promote its accumulation and increase endolymphatic pressure [129,150]. This mechanism explains why dietary interventions, although they do not directly alter plasma sodium concentration, may have a clinically relevant impact on the frequency and severity of vertigo attacks, as demonstrated in multiple clinical and experimental studies.

A second major pathophysiological axis through which non-genetic factors influence MD is the immunoinflammatory pathway. The endolymphatic sac is recognized as an immunologically active structure capable of responding to systemic inflammatory stimuli. Autoimmunity, allergies, and latent viral infections may induce chronic immune activation, with the production of proinflammatory cytokines, increased vascular permeability, and dysfunction of the blood–labyrinth barrier [58,97,103]. Allergic reactions, via the release of vasoactive and immunological mediators, may serve as triggers for acute episodes, which could account for the symptomatic improvement observed in a subset of patients following allergy-targeted interventions or elimination diets [80].

Non-genetic factors also exert a significant impact on inner ear microcirculation. Cochleovestibular structures have a high metabolic demand and low tolerance to hypoxia, making them extremely sensitive to fluctuations in blood flow. Conditions such as migraine, hypothyroidism, obstructive sleep apnea, as well as caffeine or alcohol consumption, may impair vascular autoregulation, inducing vasoconstriction, hypoperfusion, and oxidative stress [55,142,146]. These changes compromise the function of sensory cells and ionic transport systems, thereby amplifying endolymphatic instability. The association between MD and migraine suggests the presence of shared vascular and neuroinflammatory mechanisms, which may explain the clinical overlap and the difficulties in differential diagnosis between these entities [46].

Circadian rhythm disruption, sleep deprivation, and chronic stress activate the hypothalamic–pituitary–adrenocortical axis, increasing cortisol and catecholamine levels [139]. These changes may affect both vascular regulation and fluid balance through their effects on vasopressin secretion. Elevated or inappropriate levels of antidiuretic hormone have been associated with increased endolymph production and EH, suggesting that sleep disturbances may indirectly contribute to the exacerbation of MD symptoms. In this regard, the relationship between MD and sleep disorders is bidirectional: vestibular symptoms impair sleep quality, while insufficient or disturbed sleep amplifies the pathophysiological mechanisms of the disease [140].

Experimental and clinical data suggest that excessive activation of the vasopressin system may reduce the drainage capacity of the endolymphatic sac and increase endolymphatic pressure, thereby amplifying the mechanical instability of vestibulo-cochlear structures. In this context, vasopressin represents an important mediator through which various non-genetic factors—such as fluid restriction, variations in sodium intake, stress, sleep disturbances, or activation of the hypothalamic–pituitary–adrenal axis—can directly influence the pathophysiological mechanisms of MD. For example, increased vasopressin secretion induced by stress or sleep deprivation may contribute to endolymph accumulation, while rapid fluctuations in fluid intake may lead to acute hormonal changes, favoring the occurrence of vertigo episodes. Thus, vasopressin can be considered a true “integration node” between environmental factors and inner ear homeostasis, translating systemic influences into local changes in endolymph volume and pressure. This perspective supports the hypothesis that the stability of the neuroendocrine environment is essential for disease control and provides a mechanistic explanation for the effectiveness of interventions targeting regularity in fluid intake, diet, and circadian rhythm.

With respect to the role of hydro-electrolytic imbalance and dietary factors, the observations of our manuscript are consistent with the results reported by Tyrrell et al. [3,151,152], who demonstrated that daily variations in sodium and fluid intake are more clinically relevant than absolute consumption, due to their impact on vasopressin and aldosterone secretion. This study supports the idea that hormonally mediated instability induced by extrinsic factors may promote fluctuations in endolymphatic volume, providing a coherent explanation for attack triggering. In addition, Foster and Breeze [25] emphasized that dietary restriction influences not only EH but also vestibular neuronal excitability, suggesting an additional neurophysiological mechanism that complements data regarding the effects of caffeine and alcohol.

The immunological component is further supported by broader evidence of systemic immune activation in MD. Ruckenstein et al. [66], and later Greco et al. [64], demonstrated elevated levels of proinflammatory cytokines (IL-1β, TNF-α) in patients with active MD, suggesting that chronic inflammation may impair endolymphatic sac function independently of the initial etiology. This association points toward the existence of a shared immunopathological substrate rather than a simple coincidence. Importantly, autoimmune-driven inflammation provides a mechanistic explanation for the fluctuating nature of MD symptoms and for the bilateral progression observed in some patients [58,79,81,86]. From a clinical perspective, this association underscores the importance of screening for autoimmune comorbidities in patients with MD, particularly in cases of bilateral involvement, atypical progression, or poor response to standard therapy.

The role of allergies is supported by the studies of Derebery et al. [84,97], Keles et al. [89] and Pan et al. [88], which demonstrated an increased prevalence of allergic rhinitis and food hypersensitivities in patients with MD. The results were also reported by Weinreich et al. [90] and Xu et al. [91] in their studies. These studies propose a pathophysiological mechanism based on the release of histamine and leukotrienes, affecting labyrinthine vascular permeability and endolymphatic pressure. Moreover, allergic inflammation may synergistically interact with autoimmune mechanisms, amplifying immune activation at the level of the endolymphatic sac. Although causal relationships are difficult to establish, the consistency of observational data suggests that allergic diseases may function more as disease-modifying factors rather than as a primary cause of MD [117].

Migraine-associated vascular instability may predispose the inner ear to transient ischemia or altered perfusion, exacerbating EH-related symptoms. Furthermore, migraine-associated neuroinflammation and cortical hyperexcitability may lower the threshold for vestibular symptom perception, amplifying the clinical burden of MD. This overlap complicates differential diagnosis but also supports the concept of a shared central-peripheral dysfunction spectrum rather than strictly distinct disease entities [46,87]. The link between migraine and MD is further supported by the studies of Neff et al. [44] and van Esch et al. [51], which proposed the existence of a migraine–MD spectrum mediated by neurovascular dysfunction and brainstem hypersensitivity. These findings suggest that non-genetic factors such as stress, sleep deprivation, or hormonal fluctuations may trigger both migraine attacks and Ménière episodes via shared central mechanisms, reinforcing observations regarding the role of neuroendocrine dysregulation.

Viral mechanisms have been explored by Gacek et al. [153] and Welling et al. [106], who identified viral DNA (HSV-1) in the vestibular ganglion of patients with MD. These studies suggest that viral reactivation, facilitated by stress or transient immunosuppression, may induce local inflammation and vestibular dysfunction. Although the evidence remains circumstantial, viral mechanisms are biologically plausible and consistent with the episodic, recurrent-remitting course of MD observed in many patients. Further research is needed to clarify whether antiviral or immunomodulatory interventions could benefit specific patient subgroups.

Sleep disturbances further modulate disease expression through neuroendocrine dysregulation, intermittent hypoxia, and autonomic imbalance. The association between MD and obstructive sleep apnea highlights the vulnerability of the inner ear to hypoxic and microvascular stress, reinforcing the importance of sleep assessment and management [142]. Finally, data regarding sleep disturbances are supported by the observations of Eckhard et al. [39], who demonstrated that circadian rhythm disruptions can influence the expression of genes involved in ionic transport and inflammation in the inner ear. This perspective provides a bridge between non-genetic factors and epigenetic regulation, suggesting that chronic exposures may alter the functional expression of pathways involved in EH without changing the underlying genetic sequence.

Estrogen and progesterone influence vascular tone, fluid retention, and immune responses, all of which are relevant to inner ear homeostasis. Fluctuations in these hormones may partially explain the higher prevalence and symptomatic variability observed in women, particularly during periods of hormonal instability [119]. Glycemic instability can impair microcirculation, increase oxidative stress, and alter endothelial function, thereby affecting inner ear perfusion and fluid balance. These mechanisms provide a rationale for dietary strategies aimed at stabilizing metabolic and hormonal fluctuations as part of MD management [133].

Differences in healthcare access across geographic regions and socioeconomic levels also influence disease detectability. Rural populations or those with limited resources tend to be underrepresented in official statistics, potentially creating the false impression of lower prevalence in these groups. Moreover, the lack of standardized audiometric and vestibular assessments reduces the reliability of inclusion criteria, as some studies rely solely on clinical diagnosis without objective investigations [113].

Despite these numerous limitations, existing epidemiological studies provide useful reference points regarding the distribution of MD in the population and may suggest possible differences related to age, sex, race, or socioeconomic status. However, interpretation of these results requires substantial caution and a deep understanding of potential methodological biases affecting reported data. Strengthening future studies will require implementing standardized diagnostic definitions, collecting longitudinal data, improving the accuracy of medical coding, and more clearly delineating at-risk populations. Only through such measures can robustly estimates of the true prevalence and incidence of MD be obtained, and its overall epidemiological impact clarified [113].

Given the major role of non-genetic factors in modulating the pathophysiological mechanisms of MD, strategies to limit their adverse effects should focus not only on symptom control but, more importantly, on stabilizing the biological environment of the inner ear. One of the most effective intervention strategies focuses on optimizing hydro-electrolytic balance. Controlling sodium intake and daily variations in fluid consumption is not intended to directly alter plasma sodium levels but rather to reduce hormonal fluctuations involved in regulating endolymphatic volume, particularly within the renin–angiotensin–aldosterone system and vasopressin pathways [99]. Stabilizing these axes limits hyperactivation of endolymphatic secretion mechanisms and promotes more efficient functioning of absorption systems at the endolymphatic sac.

Controlling systemic inflammation represents another essential avenue in limiting the effects of non-genetic factors. Identification and treatment of associated autoimmune conditions, particularly autoimmune thyroid disease, can reduce chronic immune activation affecting the endolymphatic sac. Appropriate replacement therapy in hypothyroidism, along with monitoring inflammatory markers, can help normalize vascular permeability and restore endolymphatic drainage function. Immunomodulatory strategies, although not yet standardized, may represent a future therapeutic direction for selected patients. Similarly, recognizing the role of respiratory or food allergies allows the implementation of allergen avoidance strategies or pharmacological control of allergic responses, reducing the release of proinflammatory and vasoactive mediators that can precipitate MD attacks [154,155].

Optimizing cardiovascular parameters, including blood pressure, lipid profile, and endothelial function, contributes to maintaining adequate inner ear blood flow [156]. In this context, reducing the consumption of vasoconstrictive substances such as caffeine and nicotine may improve vascular autoregulation and prevent episodes of labyrinthine hypoperfusion. Furthermore, identifying patients with comorbid migraine and implementing targeted management can reduce the impact of shared neurovascular mechanisms, limiting symptomatic overlap and vertigo exacerbation [154,155]. Treating obstructive sleep apnea, insomnia, or other sleep disorders can reduce neuroendocrine instability and prevent recurrent exacerbations of vestibular symptoms. Similarly, stress-reduction strategies can decrease hyperactivation of the hypothalamic–pituitary–adrenal axis, limiting the negative effects of cortisol on microcirculation and immune balance [138,140].

Non-genetic factors should not be viewed as isolated disease determinants but as modulators of an already vulnerable system. They explain the episodic nature of the disease, interindividual variability, and unequal treatment responses, contributing to the transformation of latent susceptibility into clinically manifest disease. Their clinical significance lies in the fact that they represent potentially modifiable therapeutic targets.

Future research should focus on identifying distinct MD endophenotypes, clarifying causal pathways, and developing personalized therapeutic strategies. From a clinical perspective, comprehensive assessment and management of non-genetic factors should be considered essential components of care for patients with MD. Such an approach offers the greatest potential to improve symptom control, reduce disease burden, and optimize long-term outcomes for patients with Ménière’s disease.

Overall, the current literature supports the role of non-genetic factors in Ménière’s disease; however, it is characterized by a lack of methodological standardization and a predominant reliance on observational studies. This situation requires a cautious interpretation of findings and underscores the need for rigorous future research based on well-defined cohorts, validated biomarkers, and prospective or interventional study designs capable of clarifying causal relationships and supporting the development of personalized therapeutic strategies.

## Figures and Tables

**Table 1:** The main endophenotypes in Ménière’s disease [28,30].

Frejo et al. 2016 [30]	Frejo et al. 2017 [28]
GROUP I The first group included 50% of the patients and defined by unilateral onset with sensorineural hearing loss and progression to bilateral involvement over time, without associated migraine or autoimmune disorders.	GROUP I The first group included 53% of the patients and was defined by sporadic MD, without migraine or autoimmune comorbidities.
GROUP II The second group included 17% of the patients, defined by bilateral sensorineural hearing loss without migraine or autoimmune comorbidities.	GROUP II The second group included patients with MD and migraine and represented 15% of the patients.
GROUP III The third group included familial cases of MD, characterized by a strong genetic background and represented 13% of the patients.	GROUP III The third group included patients with familial MD and represented 13% of the patients.
GROUP IV The fourth group was associated with migraines and represented 12% of the patients.	GROUP IV The fourth group was defined by the presence of an autoimmune disease and represented 11% of the patients.
GROUP V The fifth group included 11% of the patients and presented autoimmune comorbidities, such as autoimmune thyroiditis, rheumatoid arthritis, or psoriasis.	GROUP V The fifth group presented hearing loss that occurred months or years before the onset of vertigo (the so-called delayed MD), like the second largest group among patients with unilateral MD and represented 8% of the patients.

**Table 2:** Nutritional recommendations in MD [99,126,127,128,129,130,131,132,133,134,135].

Foods Recommended in Ménière’s Disease		Foods Not Recommended in Ménière’s Disease
VEGETABLES	Vegetables are essential due to their low sodium content, fiber intake, and anti-inflammatory role. Fresh or frozen vegetables are recommended, prepared simply without added salt, such as in soups or purées. Are recommended: Green vegetables: spinach, broccoli, lettuce, zucchini Root vegetables: carrot, beetroot, parsnip, parsley root, celery Cruciferous vegetables: cauliflower, cabbage Fresh vegetables: tomatoes, cucumbers, white potatoes, sweet potatoes, onions, garlic Dried legumes: lentils, chickpeas, beans	Are not recommended: Canned vegetables Pickled vegetables Instant soups Vegetables preserved in brine Stock cubes/bouillon cubes
FRUITS	Fruits provide antioxidants and help maintain metabolic balance. Are recommended: Apples, pears, plums, grapes, apricots, peaches Berries (blueberries, raspberries) Citrus fruits Bananas (in moderation, due to their potassium content)	Are not recommended: Candied fruits Fruits canned in syrup Industrial fruit juices Compotes with added sugar and additives Industrial jam or marmalade
MEAT PRODUCTS	Proteins are important for blood glucose stability and for preventing metabolic fluctuations. They should be prepared without added salt, by boiling, baking, or steaming. Are recommended: Lean meat: chicken, turkey, rabbit Fresh fish (salmon, cod, trout, mackerel) Eggs	Are not recommended: Processed meats: sausages, ham, salami, bacon Smoked or preserved meat Smoked fish, fish preserved in brine, roe, preserved or raw seafood Red meat: pork, beef
DAIRY	Are recommended: Skimmed or semi-skimmed milk Plain yogurt Fresh unsalted cheese Unsalted cottage cheese Kefir, sana	Are not recommended: Aged cheeses (brie, feta, cedar, camembert, etc.) Processed cheeses Salted cream
GRAINS	Whole grains help maintain stable blood sugar levels and prevent reactive hypoglycemia. Are recommended: Brown rice, quinoa Oats, rye Whole-grain bread with low sodium content Plain whole-grain pasta	Are not recommended: Processed white bread Pretzels, salted biscuits Commercial pastries White pasta
FATS	Unsaturated fats have anti-inflammatory effects and provide vascular protection. Are recommended: Extra virgin olive oil Canola oil Avocado Unsalted nuts and seeds Fatty fish	Are not recommended: Margarin Hydrogenated fats Salty chips and snacks Pizza, hamburgers, French fries, fast-food products
SUGAR/ SWEETS	Are recommended: Homemade sweets with reduced sugar Fruit as dessert Replacing refined sugar with natural sweeteners	Are not recommended: Chocolate Commercial pastries Candy Products with refined sugar
DRINKS	It is important to maintain a consistent fluid intake, avoiding both dehydration and sudden excessive consumption. Are recommended: Still water Caffeine-free teas Herbal infusions (chamomile, mint, ginger etc)	Caffeine has a stimulating effect on the nervous system and may worsen tinnitus and vertigo through its vascular and neuro-excitatory effects. Alcohol can alter blood flow in the inner ear and affect endolymphatic pressure, being associated with worsening of symptoms. Are not recommended: Coffee Energy drinks Carbonated soft drinks Black or green tea
SALT	Are recommended: Salt-free spices Aromatic herbs (basil, oregano, thyme) Lemon juice Balsamic vinegar	Are not recommended: Table salt, iodized salt Commercial seasonings containing sodium Monosodium glutamate (MSG) Preservatives and flavour enhancers Commercial Asian foods Industrial sauces (soy sauce, teriyaki) Frozen foods Tomato paste, sauces
POTASSIUM	Potassium helps maintain fluid and electrolyte balance and can counteract the effects of sodium. Bananas Sweet potatoes Spinach Avocado Vegetables	

## Data Availability

No new data were created or analyzed in this study.

## Abbreviations

The following abbreviations are used in this manuscript:

AAO-HNS	American Academy of Otolaryngology–Head and Neck Surgery
AD	Autosomal dominant
AIED	Autoimmune inner ear disease
ATP1A2	ATPase Na+/K+ Transporting Subunit Alpha 2
ATP6V0E	ATPase H+ Transporting V0 Subunit E1
CACNA1A	Calcium Voltage-Gated Channel Subunit Alpha1 A
DRD1	Dopamine Receptor D1
EAONO	European Academy of Otology and Neurotology
EH	Endolymphatic hydrops
FMD	Familial Ménière’s disease
FHM	Familial hemiplegic migraine
GABRP	Gamma-Aminobutyric Acid Type A Receptor Subunit Pi
HCFC1	Host Cell Factor C1
HLA	Human leukocyte antigen
HRH2	Histamine Receptor H2
IL-1β	Interleukin-1 beta
IL-6	Interleukin-6
KCNMB1	Potassium Calcium-Activated Channel Subfamily M Regulatory Beta Subunit 1
KCNIP1	Potassium Voltage-Gated Channel Interacting Protein 1
LYP	Lymphoid tyrosine phosphatase
MD	Ménière’s disease
MICA	MHC class I chain-related gene A
MSG	Mmonosodium glutamate
NFKB1	Nuclear Factor Kappa B Subunit 1
OSA	Obstructive sleep apnea
PTPN22	Protein Tyrosine Phosphatase Non-Receptor Type 22
SCN1A	Sodium Voltage-Gated Channel Alpha Subunit 1
SLC34A1	Solute Carrier Family 34 Member 1
SMD	Sporadic Ménière’s disease
T3	Triiodothyronine
T4	Thyroxine
TLR10	Toll-like receptor 10
TNF-α	Tumor necrosis factor
